# Heat disproportionately kills young people: Evidence from wet-bulb temperature in Mexico

**DOI:** 10.1126/sciadv.adq3367

**Published:** 2024-12-06

**Authors:** Andrew J. Wilson, R. Daniel Bressler, Catherine Ivanovich, Cascade Tuholske, Colin Raymond, Radley M. Horton, Adam Sobel, Patrick Kinney, Tereza Cavazos, Jeffrey G. Shrader

**Affiliations:** ^1^ Center on Food Security and the Environment, Stanford University, Palo Alto, CA, USA.; ^2^ Global Policy Laboratory, Stanford University, Palo Alto, CA, USA.; ^3^ Center for Environmental Economics and Policy, Columbia University, New York, NY, USA.; ^4^ School of International and Public Affairs, Columbia University, New York, NY, USA.; ^5^ Climate School, Columbia University, New York, NY, USA.; ^6^ Department of Earth and Environmental Sciences, Columbia University, New York, NY, USA.; ^7^ Department of Earth Sciences, Montana State University, Bozeman, MT, USA.; ^8^ Joint Institute for Regional Earth System Science and Engineering, University of California, Los Angeles, Los Angeles, CA, USA.; ^9^ NASA Jet Propulsion Laboratory, La Cañada Flintridge, CA, USA.; ^10^ Department of Applied Physics and Applied Mathematics, Columbia University, New York, NY, USA.; ^11^ School of Public Health, Boston University, Boston, MA, USA.; ^12^ Department of Physical Oceanography, CICESE, B.C., Mexico.

## Abstract

Recent studies project that temperature-related mortality will be the largest source of damage from climate change, with particular concern for the elderly whom it is believed bear the largest heat-related mortality risk. We study heat and mortality in Mexico, a country that exhibits a unique combination of universal mortality microdata and among the most extreme levels of humid heat. Combining detailed measurements of wet-bulb temperature with age-specific mortality data, we find that younger people are particularly vulnerable to heat: People under 35 years old account for 75% of recent heat-related deaths and 87% of heat-related lost life years, while those 50 and older account for 96% of cold-related deaths and 80% of cold-related lost life years. We develop high-resolution projections of humid heat and associated mortality and find that under the end-of-century SSP 3–7.0 emissions scenario, temperature-related deaths shift from older to younger people. Deaths among under-35-year-olds increase 32% while decreasing by 33% among other age groups.

## Teaser

Analysis of temperature exposure and microdata finds the majority of heat-related deaths are among people under 35 years old.

## INTRODUCTION

Historically, temperature exposure has caused a large number of premature deaths (*1*–*3*). Heat-related mortality is expected to increase under climate change (*4*–*27*). As the evidence base has grown, multiple studies have found that the elderly are especially vulnerable to heat (*6*, *11*, *14*, *17*, *18*, *28*, *29*). Furthermore, many other studies have expressed particular concern for joint heat and humidity extremes, given the importance of perspiration for human thermoregulation (*30*–*36*).

In this study, we explore the relationship between humid heat and mortality in Mexico, a country that exhibits a unique combination of rich, age-specific, universal mortality microdata and among the most extreme historical humid heat exposures. We find that historically, the majority of heat-related mortality in Mexico has been concentrated among younger people: 75% of heat-related deaths and 87% of heat-related lost life years occur among those under 35 years old. By contrast, the vast majority of cold-related mortality is concentrated among older people: 98% of cold-related deaths and 90% of cold-related lost life years occur among those over 35, with the majority of cold-related deaths occurring among individuals older than 70 years. We then develop projections of humid heat and associated outcomes to assess the future implications of these findings. As in other studies, we find that climate change is expected to increase heat-related mortality while decreasing cold-related mortality. However, we uncover an important source of future climate-driven inequality: The disproportionate impact of heat and cold across age groups reallocates the temperature-related mortality burden from the elderly (who are more affected by cold) to the young (who are more affected by heat). This has important implications for understanding the distributional impacts of climate change and for developing effective policies to adapt to these impacts.

## METHODS

Our insights into the effect of humid heat across the life span result from a combination of four elements: (i) station-level wet-bulb temperature estimates; (ii) high-quality, age-specific, population-wide mortality microdata; (iii) a statistical method that resolves age-specific heterogeneity in temperature vulnerability; and (iv) realistic, granular projections of humid heat under climate change across our study area.

First, we study the effect of wet-bulb temperature on mortality. While multiple metrics exist to measure humid heat stress (*37*), wet-bulb temperature has been identified as an important metric for understanding the impact of heat on human health because it accounts for the critical role of sweat evaporation—the primary mechanism by which the human body cools itself—in maintaining homeostasis under heat exposure (*36*, *38*). Under high humidity, sweating efficiency decreases (*37*, *39*, *40*). When ambient wet-bulb temperature exceeds human skin temperature (at around 35°C), humans can no longer dissipate heat into the environment and are thus physically incapable of survival when exposed for a sufficient length of time (*30*, *32*, *33*). In practice, experimental evidence has shown that heat stress can become uncompensable at wet-bulb temperatures of 31°C or lower (*34*, *35*). Under high-emissions scenarios, increasing humid heat stress is projected to cause some regions to become uninhabitable for parts of the year without artificial cooling (*33*). Despite the importance of both heat and humidity for human thermoregulation, most empirical studies on temperature-related mortality have focused on dry-bulb temperature, which does not account for humidity. Hundreds of papers have been written on the mortality impact associated with dry-bulb temperature (*4*, *8*). One review found only nine papers that assessed the role of humid heat on mortality (*31*), and there remain important gaps in our understanding of the population-wide health impacts of humid heat (*36*).

Second, our study leverages precise historical data on both mortality and temperature exposure. Mexico’s high-quality vital statistics microdata includes a record of each death occurring in the country since 1998. Crucially, these microdata also contain information on age at death, allowing us to assess age-specific heterogeneity in the relationship of heat and mortality with more precision than prior literature, which has focused on broader age groups or on effects only among the elderly (*6*, *12*, *36*, *41*, *42*). The dataset spans the 22 years from 1998 to 2019. We choose to end our study period in 2019, before the COVID-19 pandemic. The data contain information on the day and municipality—Mexico’s second-order administrative unit, numbering around 2400 across the country—of occurrence of 13.4 million deaths over more than 21 million municipality-days. We combine these records with station-level, subdaily measurements of dry-bulb temperature, humidity, and air pressure, which we use to develop estimates of local daily mean wet-bulb temperature (*43*). This is important because we find evidence that weather reanalysis data products such as ERA5-Land do not reproduce the most extreme humid heat events observed by Mexico’s station network (see fig. S13). Mexico’s heterogeneous climate and rich public vital statistics records make it an ideal setting for determining the impact of humid heat exposure on premature mortality. Mexico is one of the most climatically diverse countries in the world, with the fourth largest number of Köppen climate zones (see fig. S14). It is located in the subtropics and tropical regions, has a wide variety of elevations, is located between two oceans, and experiences substantial seasonal variation, including the North American monsoon. The low correlation between air temperature and humidity observed throughout many areas of Mexico (*36*) allows extreme dry and humid heat events to take place on separate days within the same location, facilitating the investigation of the distinct impacts of these two extremes. Most existing studies have assessed temperature-mortality relationships in cooler, higher-income countries (*30*, *36*) that have rarely if ever experienced humid heat extremes. Mexico, by contrast, has experienced among the highest wet-bulb temperatures ever recorded, particularly in coastal regions (*32*). Substantial populations are also exposed to these diverse climates across the country (see fig. S14).

Third, we estimate an age-specific exposure relationship between excess mortality and daily average wet- and dry-bulb temperatures. Our empirical model leverages current best practices to isolate causal impacts of temperature on excess mortality (*1*, *6*). We investigate effects over a set of distributed lags to capture the dynamic effects of temperature on health, including harvesting—when “deaths are occurring only a few days early among persons who were already dying” (*44*)—and delayed mortality responses. Our model flexibly captures differences in impacts from cold-, moderate-, and hot-temperature exposures and includes control variables to account for potential confounders, including seasonality and time trends. We identify effects on the basis of otherwise random changes in weather across days within a given municipality, such that a municipality experiencing mild weather acts as the “control group” for itself during more extreme weather, eliminating confounding spatial variation. Last, we flexibly adjust for daily precipitation to ensure that the effects of temperature are not operating via rainfall. Our statistical model allows the minimum mortality temperature to vary by age group. We find that different age groups experience minimum mortality at substantially different temperatures: Individuals in their 70s experience minimum mortality at temperatures nearly 10°C higher than individuals in their 20s for both dry-bulb and wet-bulb temperatures (see fig. S11). See section A.2 for further details on the model and estimation procedure.

Last, we develop fine-scale projections of dry and humid heat through the end of the century to project changes in mortality across age groups as the climate warms. We retrieve statistically down-scaled temperature, humidity, and precipitation projections through the end of the century (*45*) across the greenhouse gas emissions associated with four Shared Socioeconomic Pathways (SSPs) (*46*); calculate wet-bulb temperature; and bias correct the dry-bulb temperature, wet-bulb temperature, and precipitation projections against historical station and reanalysis data (*47*, *48*) using percentile mapping. This approach allows us to best match the spatial distribution of the available human health data, as well as to capture the variability in climate and terrain throughout Mexico, essential to reproducing dry and humid heat extremes (*49*). For additional details, see section A.1.2.

## RESULTS

**Fig. 1.**
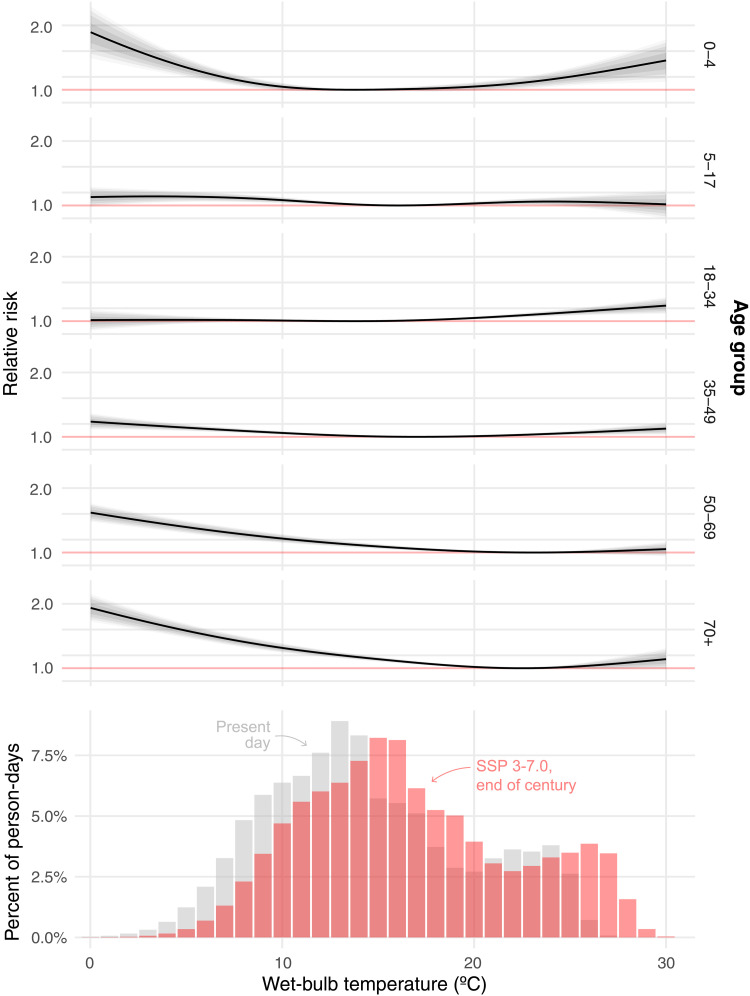
Relationships between mortality risk and exposure to wet-bulb temperature by age group in Mexico. The top panels show the change in relative mortality risk (*y* axis) caused by exposure to 1 day of the indicated average daily wet-bulb temperatures (*x* axis) across age groups. The bottom panel shows the distribution of daily average wet-bulb temperatures in Mexico throughout our sample period as well as the ensemble mean of projected temperature distribution at the end of the century (2083–2099) under the SSP 3–7.0 greenhouse gas (GHG) emissions scenario. Shaded bands around the functions in the top panels indicate 95, 90, 80, and 50% confidence intervals. Absolute changes in mortality for both wet- and dry-bulb temperature are shown in fig. S1, and coefficient estimates for absolute mortality changes are shown in table S1.

**Fig. 2.**
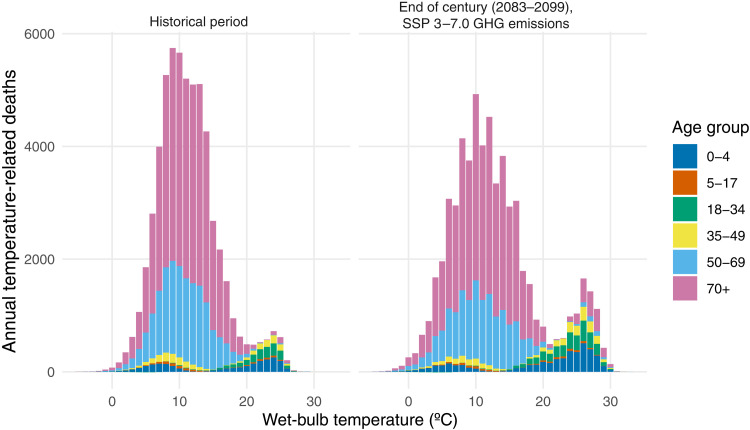
Historical and projected annual temperature-related deaths in Mexico. The panels show average annual temperature-related deaths resulting from exposure to days with the average wet-bulb temperatures shown on the *x* axis during the historical period (**left**) and at the end of the century (2083–2099) under the SSP 3–7.0 GHG emissions scenario (**right**) across six age groups in Mexico. The figure shows mean projected deaths; see Fig. 3 for projections with uncertainty.

**Fig. 3.**
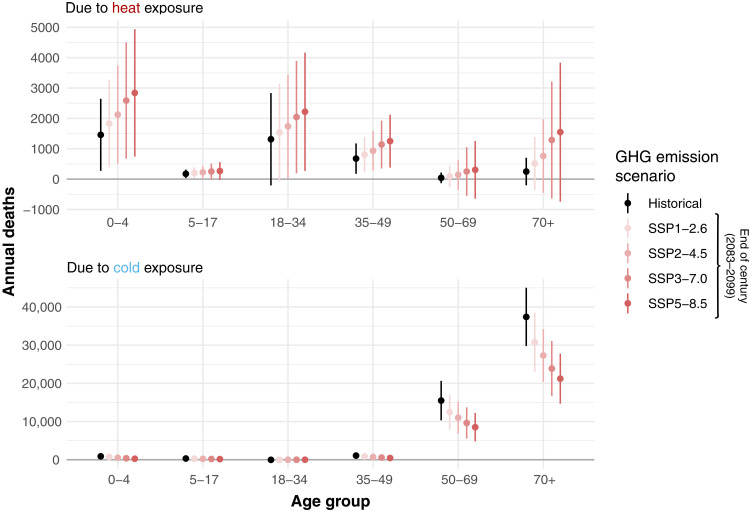
Historical and projected annual deaths due to heat and cold exposure by age group. The figure depicts the average annual number of deaths attributed to heat (**top**) and cold (**bottom**) exposure in Mexico historically and under wet-bulb temperatures prevailing at the end of the century in four GHG emissions scenarios. Whiskers above and below each estimate depict 95% confidence intervals net of both econometric and climate uncertainty. Note that the range of the *y* axis in the bottom panel is roughly eight times the range of the *y* axis in the top panel.

Figure 1 shows the effect of exposure to a single day at the indicated wet-bulb temperature on mortality risk for different age groups. For instance, the under-5 exposure-response function implies that when an individual under 5 years of age experiences 1 day with average wet-bulb temperature of 27°C, their risk of mortality increases by 45% relative to if they had experienced 1 day with an average wet-bulb temperature of 13°C. (For policy-makers, numerical values for the estimated additional number of deaths per person for different temperature exposures are shown in table S1.) Figure 2 (left) combines the age-specific vulnerability to heat and cold (shown in Fig. 1, top) along with the frequency with which those temperatures occur (shown in Fig. 1, bottom) to quantify the total annual number of temperature-related deaths associated with exposure to temperature broken down into 1° temperature bins and broken out by age group during our historical data period. In Fig. 3, points labeled “Historical” aggregate these data to quantify the total annual number of heat- and cold-related deaths by age group. These values combine age-specific vulnerability to heat and cold (shown in the top panels of Fig. 1) with the frequency with which those temperatures occur (shown in the bottom row of Fig. 1).

Consistent with past literature on temperature-related mortality in Mexico (*28*, *50*), we find that cold is historically associated with more deaths than heat across the whole population: Cold causes 14 times more deaths than heat, as shown in Fig. 3. However, this masks important heterogeneity across age groups. While cold-related mortality is concentrated among the old, heat-related mortality is concentrated among the young. For individuals under 35, heat causes 2.6 times more deaths than cold (Fig. 3, top), whereas for individuals 35 and older, cold causes 56 times more deaths than heat (Fig. 3, bottom). Ninety-eight percent of cold-related deaths occurred among those 35 and older, with 28% of such deaths occurring among those 50 to 70 and 68% occurring among those 70 and older. In contrast 75% of heat-related deaths occurred among under-35-year-olds (the distributions of these proportions are shown in fig. S5). This contrasts with the previous literature, which has found that both cold- and heat-related mortality impacts are concentrated among elderly people.

**Fig. 4.**
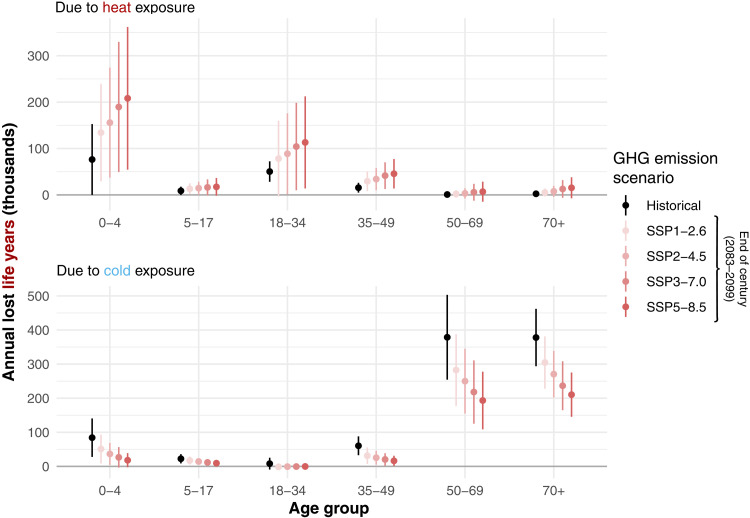
Historical and projected annual lost life years due to heat and cold exposure by age group. This figure mirrors Fig. 3 but with the outcome as lost life years, rather than deaths. Potential remaining life years are taken from the UN World Population Prospects 2022 and are aggregated to time-invariant age group values by taking a population-weighted average across single age bins and years. The figure depicts the annual number of lost life years attributed to heat (**top**) and cold (**bottom**) exposure in Mexico historically and under wet-bulb temperatures prevailing at the end of the century in four GHG emissions scenarios. Whiskers above and below each estimate depict 95% confidence intervals net of both econometric and climate uncertainty.

When considering lost life years, which accounts for the fact that younger individuals have on average more remaining life than older individuals, the outsized impact of heat on younger age groups becomes even more pronounced (Fig. 4): Those under 35 years old account for 87% of life years lost because of recent heat exposure, whereas those 50 and older account for 80% of life years lost to recent cold exposure.

We find that these results on the concentration of the heat-related mortality burden among the young and the cold-related mortality burden among the old are robust whether we use wet- or dry-bulb temperature as our metric of exposure (as shown in figs. S1 to S5). Though, nearly all historical exposures—even in our context—are below theoretically uncompensable humid heat levels (*34*, *51*).

The right panel of Fig. 2 and the red points and whiskers in Fig. 3 show our projections for the number of annual deaths at the end of the century broken down by age group. These projected deaths do not account for potential future adaptation or population changes but rather describe the effect of projected future temperatures on mortality given historical socioeconomic, institutional, and adaptation conditions. Climate change is projected to cause more heat-related mortality and less cold-related mortality across all age groups. Cold-related mortality continues to be concentrated among individuals 35 and older—with the impact especially pronounced on individuals 70 or older—while heat-related mortality continues to be concentrated among individuals under 35 years old. However, as hot days become more frequent and cold days become less frequent, the overall temperature-related mortality burden shifts toward the young and away from the old. Older individuals continue to suffer disproportionately from cold-related mortality, but cold days are comparatively less frequent. Those under 35 suffer disproportionately from increasing heat, with premature mortality especially concentrated in the under-5 and 18 to 34 age groups.

Figure 3 and fig. S6 show the projected percent change in age group temperature-related deaths at the end of the century across four different greenhouse gas emissions scenarios, ranging from a rapid decarbonization scenario (SSP 1–2.6) to a very high emissions scenario (SSP 5–8.5). All results are relative to historical temperature-related deaths. These figures show that the age structure of mortality burdens in Fig. 2 holds more generally across low-, medium-, and high-emissions scenarios. In all scenarios, climate change shifts the risk of temperature-related mortality toward those under 35 and away from those 50 and older. Under the SSP 3–7.0 emissions scenario, we project a 32% increase in temperature-related deaths among under-35-year-olds driven by an increase in heat-related mortality and a 33% decrease among those 35 and older driven by a decrease in cold-related mortality (the distributions of these estimates of percent changes in overall temperature-related mortality are shown in figs. S6 and S7).

Previous research has shown that dry-bulb temperature-related mortality in Mexico is currently driven primarily by cold (*50*) and that under climate change, temperature-related mortality will fall in Mexico as the benefits from reduced cold outweigh the harms from increased heat (*6*). Our results present a more complicated picture: We find—consistent with prior literature—that temperature-related mortality as a whole will fall in Mexico under climate change, but when taking age-specific effects into account, we project that this will happen at the expense of younger individuals.

## DISCUSSION

The unique combination of elements in this study—station-level wet-bulb temperature estimates, granular mortality data from across the entire age distribution in a country with a wide diversity of climatic conditions, a statistical method that captures age-specific heterogeneity in temperature vulnerability, and high-resolution projections of humid heat—deepens our understanding of multiple aspects of the impact of temperature on mortality. By focusing on granular, age-specific temperature-mortality impacts, our study contributes to the existing literature that has usually focused on mortality irrespective of age (*1*, *8*, *52*), across broader age groups (*6*, *42*), or on the elderly alone (*12*, *13*). In particular, in our setting, we find that while individuals 35 and older suffer the vast majority of the cold-related mortality burden, those younger than 35 suffer most of the heat-related mortality burden. In addition, we identify a source of climate-driven inequality that has not been identified in previous studies: Across all future emissions scenarios, we find that climate change causes the temperature-related mortality burden to shift away from the elderly toward the young. Given that temperature-related mortality is projected to be the largest single source of climate damages (*53*, *54*), the disproportionate burden of this impact on the young is likely an important source of future climate-driven inequality.

Prior research has discussed multiple reasons that older individuals are vulnerable to cold temperatures. These reasons are physiological, behavioral, and social. First, the elderly exhibit lower shivering temperature thresholds (*55*) and have substantially lower levels of brown adipose tissue (key for nonshivering thermogenesis) (*56*). Second, a relatively large proportion of elderly individuals have preexisting medical conditions or attendant respiratory illnesses that can be contributing factors in cold-related mortality (*57*). Third, elderly individuals are increasingly living alone, making it more difficult for them to access public health resources during extreme weather events, and they experience higher rates of loneliness, which is correlated with worse cardiovascular health (*58*). Fourth, energy poverty—spending a large fraction of income on energy—can be particularly acute for elderly individuals. Mexico has both a high rate of energy poverty and a high prevalence of credit constraints that might prevent adoption of protective but energy-intensive home heating (*59*). In this study, we indeed find that the elderly are, in terms of absolute mortality impacts, far more vulnerable to cold than other age groups (fig. S1). We find that the vast majority of cold-related mortality is concentrated in those 50 and older, as shown in the top panels of Fig. 2.

However, we find that young people are particularly vulnerable to heat: The majority of heat-related deaths are concentrated in those under 35, and those under 35 are overrepresented in heat-related deaths relative to their fraction of the population despite their far lower background crude death rate (fig. S5). Our finding that children younger than 5 years old are especially vulnerable to heat (Fig. 1) is directionally consistent with some prior work, although we find particularly acute effects. Multiple potential mechanisms may contribute to this result. First, infants have a higher body surface area-to-body weight ratio than adults, which means that they gain heat more rapidly and are more susceptible to overheating; infants also have a less developed thermoregulatory system (exhibiting reduced sweating), which means that they are not as efficient at regulating their body temperature (*60*). Second, very young children have less well-developed immune systems, making them more vulnerable to climate-related infectious diseases including vector-borne diseases and diarrheal diseases that might be especially affected by humid heat (*61*, *62*). Last, both infants and young children have less freedom of movement than adults and may not be able to express their discomfort or distress as easily as adults, making it more difficult for caregivers—the primary providers of child adaptation to heat exposure—to recognize and respond to their heat stress (*63*).

We also find that heat disproportionately affects those 18 to 34 years old. Younger adults are more physiologically robust to heat, but multiple behavioral, social, and economic factors can contribute to higher heat-related mortality among this age group (*41*). Younger individuals are exposed to ambient heat through sports and other recreational activities (*41*). Households with older household heads are more likely to have an air conditioner (*64*). One important channel may be occupational heat exposure: Young adults are more likely than older adults to work in outdoor occupations with minimal flexibility for precautionary action (*65*). An analysis of death certificates in Mexico shows that men of working age are more likely to have extreme weather events listed as a cause of death (*28*), though we note that death certificates typically do not capture all deaths due to extreme weather (*66*). Relatedly, we find that individuals who live in regions with higher income (itself correlated with the amount of weather-exposed occupations) are less sensitive to heat (fig. S8). Occupational exposure is likely to be an important mechanism in other countries as well given that Mexico is not out of the ordinary in terms of occupational exposure to heat. For example, during our sample period, 15% of the workforce in Mexico was used in agriculture. This is lower than the rate for other middle-income countries (30% in 2018) and all countries globally (27% in 2018) (*67*). If occupational heat exposure is indeed a driver of mortality among younger individuals, then this highlights the importance of occupational heat exposure standards for workers (*68*).

Our finding that young people in Mexico are especially vulnerable to heat may have global implications because hotter and lower-income countries—which are expected to be the most adversely affected by climate change—have among the youngest populations in the world currently and over the coming century (*69*). Figure S12 shows the current global pattern of age and wet-bulb temperature exposure. The map in the top panel breaks down countries by their most extreme wet-bulb temperatures and fraction of population younger than 35 years of age (*70*). The youngest and hottest locations in the world are concentrated in Africa, Central America, the Middle East, and portions of South and Southeast Asia. The bottom panel of fig. S12 situates Mexico in the context of the rest of the world. Mexico is near the middle of the global distribution of countries by share of population under 35, and its extreme wet-bulb temperatures are essentially only surpassed by countries in Asia. The figure also shows that historical exposure to hot wet-bulb temperature is positively correlated with the fraction of the population under 35. If our age-specific results in this study hold for other countries around the world that are younger and hotter, then existing estimates of temperature-related mortality impacts in these countries—which neither fully capture age-specific heterogeneity in the temperature-mortality relationship nor account for the impact of humid heat—may be incorrect. In past work, the lack of age-specific mortality data has been a limiting factor in exploring the age-specific temperature-mortality relationship across a large number of countries (*6*, *52*), which underscores the need for improvements in vital statistics systems, especially in the places most vulnerable to climate change.

We conclude by highlighting a few important caveats and also point to potential areas of focus for future work. Recent work has pioneered the use of both temperature and humidity for constrained joint projections (*38*, *71*, *72*). While regional and global climate models are our best tools for assessments of future heat stress risk, the relatively coarse time resolution of most model output limits the ability to project extreme values. The NASA Earth Exchange Global Daily Downscaled Projections (NEX-GDDP) dataset used in this study reports variables at a daily resolution, like many other climate model products. Given the misalignment of the diurnal cycles of temperature and humidity, using available daily mean values to calculate heat stress metrics such as wet-bulb temperature limits the accuracy of daily mean projections and is virtually impossible for daily maximum projections. These data challenges relating to the subdaily fluctuations in individual variables are even more pronounced for heat stress metrics such as wet-bulb globe temperature that incorporate additional variables relevant to the physiology of heat stress (e.g., solar insolation and wind speed) (*73*). These limitations underpin efforts to increase the temporal resolution of model data output available to end users to better represent the most extreme heat stress conditions of the future.

Our projections assume that our estimated temperature-mortality relationships will remain unchanged under future warming. There are opposing reasons why the wet-bulb temperature exposure-response functions may become either more or less severe in the future. Research on the US shows that mortality vulnerability to nonoptimal dry-bulb temperatures has decreased historically (*74*, *75*). Recent work has shown that locations with different long-run climates show different patterns of consumption responses to weather shocks (*76*). Figure S8 shows that a similar pattern holds for mortality in Mexico. However, as wet-bulb temperatures approach uncompensable levels with substantially greater frequency (*34*, *51*)—exposures of this degree are almost nonexistent in the historical record—we may learn that mortality associated with a given level of humid heat exposure is higher than existing estimates. Furthermore, our projections hold socioeconomic conditions fixed. Recently published subnational population projections for Mexico would allow future work to relax this assumption (*77*). These projections could yield higher estimates of mortality if population is trending younger in areas that are warming, or these projections could yield lower mortality estimates if the population is becoming older over time. Further estimation of the effect of income and occupational exposure could also enrich these projections and help shed light on the role of adaptation in mediating temperature-related mortality. We leave the exploration of these questions to future work.

Last, our conclusions further underscore the importance of ethical choices around monetizing the cost of premature deaths. We find that climate change is expected to shift the mortality burden away from older individuals (more affected by cold) to younger individuals (more affected by heat). Thus, the choice of whether to value life years—where premature deaths among younger individuals are considered more costly than premature deaths among old individuals—or to value all premature deaths the same becomes especially important. The US tends to value all premature deaths the same in its benefit-cost analysis (*78*), whereas UK guidance suggests that analysts can value either lives or life years (*79*). Although we do not take a stance on this difficult ethical choice, our findings further emphasize the importance of this debate for evaluations of the impact of climate change, given that we are finding that climate change is expected to shift the temperature-related mortality burden toward the young.

## Supplementary Materials

### This PDF file includes:

Supplementary Text Figs. S1 to S14 Table S1 References
