# Supplementary material for: Heat disproportionately kills young people: Evidence from wet-bulb temperature in Mexico
Source: Sci Adv. 2024 Dec 6;10(49):eadq3367. doi: 10.1126/sciadv.adq3367 (PMC11623271; doi:10.1126/sciadv.adq3367)
Supplement: Supplementary file 1 — Supplementary Text Figs. S1 to S14 Table S1 References [file sciadv.adq3367_sm.pdf]

Science Advances
AAAS

Supplementary Materials for

Heat disproportionately kills young people: Evidence from wet-bulb temperature in Mexico

Andrew J. Wilson *et al.*

Corresponding author: R. Daniel Bressler, rdb2148@columbia.edu

Sci. Adv. **10**, eadq3367 (2024)
DOI: 10.1126/sciadv.adq3367

This PDF file includes:

Supplementary Text
Figs. S1 to S14
Table S1
References

A Supplementary Materials

A.1 Data

A.1.1 Mortality, population, and life expectancy data

We collect mortality data from the Subsistema de Información Demográfica y Social of Mexico's Instituto Nacional de Estadística y Geografía. This data contains records of all recorded mortality events in Mexico since 1990. Our data period begins in 1998, when this mortality microdata first began to carry information about the day and municipality of each mortality event. Our data period ends in 2019, after which some death records are still preliminary and the COVID-19 pandemic exerts a large influence on behavior and mortality. Between 1998 and 2019, we observe in this data 13,426,931 deaths. The World Bank estimates that Mexico's all-cause crude mortality rate is around 6 deaths per 1000 people per year. Relative to a population of around 110 million during this period, this would imply total deaths of 14.5 million during our data period, giving us confidence that these mortality records are relatively complete. Across the data, each recorded mortality event also contains information on the cause of the individual's death as well as the individual's sex, education level, occupation, place of residence, and age at death. We drop death records that are missing information on the day of death, the individual's age at death, or the death location. We also drop death records reporting deaths that occurred outside of Mexico. These dropped records represent less than 0.92% of the data.

Data on administrative unit population, which is used to determine mortality rates and regression weights, is collected from IPUMS International, which consolidates and harmonizes census data for Mexico. Our study uses data from the 1990, 2000, and 2010 Mexican censuses, as well as the 2015 Intercensal Survey. Population for each administrative unit is assumed to grow at a constant rate between observations, and population growth between the 2010 Census and 2015 Intercensal Survey is assumed to remain constant through the end of our data in 2019.

Across both mortality and population data, we account for 67 municipal boundary changes occurring between 1998 and 2019 by assigning values reported for modified units to an aggregate set of 2,402 municipal units that is stable across all years of our study. We also aggregate deaths

and population across age groups: age (1) less than 5, (2) at least 5 and less than 18, (3) at least 18 and less than 35, (4) at least 35 and less than 50, (5) at least 50 and less than 70, (6) at least 70.

In this study, we sometimes report values in terms of lost life years. Our estimates for the age-dependent number of remaining life years at time of death for the average person in each age group come from the United Nations 2022 World Population Prospects, with expected remaining life years for an age group calculated as a weighted mean of the expected remaining life years of cohorts in each age classification across all sample years (i.e., expected remaining life years of those less than 5 is estimated as the expected remaining life years of age cohorts aged 0, 1, 2, 3, and 4 across every year from 1998 to 2019, weighted by the population size of each cohort). The resulting scalars are not particularly sensitive to the aggregation procedure, as potential remaining life years did not change markedly for any age group in Mexico during our data period. The scalars range from 73.32 expected remaining life years for the under-5 age group to 9.91 expected remaining life years for the 70+ age group.

A.1.2 Weather data

Our observational weather dataset is collected from the UK Met Office Hadley Centre's Integrated Surface Dataset, which consolidates observations from a global network of weather stations but performs various quality control adjustments to ensure the consistency of observations over time. The resulting dataset we use contains a set of weather metrics recorded at a sub-daily frequency (some stations report weather at an hourly frequency, but many report at three- or six-hour intervals). A map of the stations reporting data used in our analysis is shown in Figure S14.

Extreme wet-bulb temperature events at thresholds sufficiently high to impact human health may be short in duration and tightly spatially constrained [32]. Accordingly, recent literature has suggested that the spatial and temporal smoothing involved in the creation of reanalysis products often leads to underestimations of the intensity of extreme humid heat events compared to observational datasets [32]. We find that current high-resolution reanalysis data (ERA5-Land) are unable to capture humid heat extremes. This is a known limitation of reanalysis products, though recent

work has argued that reanalysis products may be suitable for studies exploring the relationship between temperature and mortality. This is not the case when such studies consider humidity, which varies more over space. Indeed, many of the most extreme humid heat events arise from short-lived intrusions of moist air above very warm seas into coastal cities on very hot days, a point emphasized in [32].

We first use the method described in [43] to approximate wet-bulb temperature from dry-bulb temperature, surface pressure, and dew point temperature at each station location. As our method requires matching station temperature records to administrative units, we then perform an adjustment to fill missing hourly dry-bulb and wet-bulb temperature observations by leveraging distributional information from nearby non-missing stations. To avoid filling missing data for stations that report infrequently or for which we do not have a sufficiently diverse historical record, we first drop from our data all stations that report fewer than 10,000 observations during the period from 1990 to 2019 (roughly 3% of hours) as well as stations that do not report more than 1000 observations across at least 10 years. For each of the remaining stations, we then determine an empirical cumulative distribution function for all non-missing observations. Next, if the data does not contain an observation for a given station at a particular hour, we determine a likely quantile for this observation using an inverse squared geodesic distance-weighted mean of all stations in Mexico that are reporting values at that hour. We then fill the missing value using the temperature at that quantile for that station. Said differently, if a station is missing data at a particular hour and nearby non-missing temperatures are on average at their 90th percentile, we set the missing value to the 90th percentile of the historical readings for that station. This method is deployed in the dataset here.

We obtain daily mean dry-bulb and wet-bulb temperatures by calculating the average of the daily minima and maxima of each metric at each station. We next determine the geodesic distance between each station and the population-weighted centroid of each administrative unit and map temperature observations to administrative units by taking the inverse squared distance-weighted mean of each temperature metric for the five nearest stations (see Figure S14 for the locations of stations used in the study). This method is similar to other papers studying temperature effects on

mortality using weather station data [74]. To determine the population-weighted centroid of each administrative unit, we use Meta's High-Resolution Population Density Maps [80]. To ensure that we are using representative weather station data to estimate exposure, we omit from our analysis municipalities whose population center of mass is more than 50 kilometers from the nearest weather station. These municipalities represent 24.83% of Mexico's population as of the date of the 2010 Census.

Precipitation data is collected from the European Centre for Medium-range Weather Forecasting Reanalysis 5 - Land (ERA5-Land) dataset (and included as a control to avoid confounding effects). Using Google Earth Engine, a daily total precipitation measure is calculated by taking a sum over the hourly values across each day at each grid cell and then taking a spatial average over each administrative unit, weighting by a gridded estimate of the time-varying distribution of population (Gridded Population of the World v4, revision 11).

A.2 Statistical model

Estimates of the effect of temperature on mortality come from fitting a mortality response function following [1]. The outcome variables—daily, location-specific mortality rates for each age group—are modeled as dynamic functions of temperature and precipitation, with additional controls for location-specific, time-varying, and seasonal confounders. Formally,

$$y_{ait} = f_a(x_{it}, \ldots, x_{it-30};\ \mathbf{B}_a) + g_a(p_{it}, \ldots, p_{it-30};\ \mathbf{\Gamma}_a) \tag{S-1}$$

$$+ \rho_{at} + \delta_{ai} \times \text{year}_t + \theta_{as} \times h(\text{doy}_t;\ \xi_a) + \varepsilon_{ait} \tag{S-2}$$

where y_{ait} is the mortality rate (deaths per person) in municipality i on date t and age group $a \in \{< 5, 5\text{–}17, 18\text{–}34, 35\text{–}49, 50\text{–}69, > 70\}$. Separate models are fit for each age group.

The main right-hand-side variable is daily average temperature—either dry-bulb or wet-bulb depending on the specification—generically denoted x_{it} in the above equation. The relationship between temperature and mortality is allowed to be nonlinear and dynamic, as captured by the function $f_a(x_{it}, \ldots, x_{it-30};\ \mathbf{B}_a)$, with $\mathbf{B}_a$ denoting the matrix of unknown coefficients to be estimated.

This function transforms temperature observations along two dimensions. In the temperature dimension, the function is a natural cubic spline over daily temperature with knots at the 10th, 50th, and 90th percentiles [81]. (For dry-bulb temperature, these knots are at approximately 14.92, 20.63, and 27.51°C; for wet-bulb temperature, they are at approximately 9.14, 15.37, and 22.80°C.) Across 22 days of distributed lags, the function is a b-spline with knots spaced equally, in log terms, across the lag period (at roughly 1, 3, and 8 days). Putting these elements together, fitting the model generates estimates of the effect of temperature on mortality at each point across the distribution of temperatures and for each of 22 days starting with with the initial day of the temperature realization. From these estimates, we calculate and report the 21-day cumulative effect of temperature on mortality, as depicted in Figure 1.

The other elements of the estimating equation are controls. Daily total precipitation, p_{it} is included in a similar way to temperature, with the cumulative effect estimated using a b-spline distributed lag. The effect of precipitation is modeled flexibly using 0th-order splines (i.e., bins) for precipitation below the 80th percentile (roughly zero precipitation), between the 80th and 90th percentiles, between the 90th and 95th percentiles, between the 95th and 98th percentiles, between the 98th and 99.5^{th} percentiles, and above the 99.5^{th} percentile (the levels of these breaks are approximately 5.31, 10.5, 15.6, 23.7, and 45.3 mm/day). Other confounders are accounted for using fixed effects, in some cases interacted with continuous controls. These controls are: date of sample fixed effects, ρ_{at}, to account for national temporal patterns, holidays, day-of-week effects, and other time-series confounders; a municipality-by-year fixed effect, $\delta_{ai} \times \text{year}_t$, to account for location-specific fixed factors such as topography, governance, and differences in access to healthcare or mortality reporting as well as secular changes in mortality rates and climate; and a state-level fixed effect, θ_{as}, interacted with a six-knot natural cubic spline over day-of-year, $h(\text{doy}_t;\ \xi_a)$, to account for state-level seasonal patterns. The term ϵ_{ait} is the remaining error term which we assume to be uncorrelated with daily temperature.

The regression is weighted by the daily municipality population (linearly interpolated from annual population counts). Standard errors are clustered at the state level to account for spatial autocovariances at the subnational level while maintaining robustness to arbitrary temporal corre-

lation patterns ($N = 32$).

Throughout, such as when we distinguish deaths from heat from those from cold or when we report the total number of temperature-related deaths, we are identifying these values relative to an age group-specific optimal temperature, or minimum mortality temperature (MMT). While semantically the MMT is simply the temperature at which mortality is minimized, the flexible functional form we use to determine the relationship between temperature and mortality leads to a corner solution for the global MMT for some age groups. To prevent this, we instead define the MMT conditionally as the temperature between the 1st and 99th percentiles of age group-specific temperature exposure at which mortality is minimized [1]. As we discuss in the main body text, MMT varies considerably by age, generally rising throughout the lifespan.

Models are fit using R software version 4.2.2 and the `fixest` package version 0.11.2 [82].

A.3 Projections

In addition to identifying historical relationships between wet-bulb temperature and mortality, we make projections of the impact of climate change on these relationships through the end of the century. The projections focus on the effect of changes in dry and moist heat, holding all other aspects of the system fixed. To make temperature-related mortality projections, we apply the age-specific exposure-response functions estimated from our statistical model to the future temperature projections described above, and assume that these functions will continue to hold in the future, as in [10]. Thus, we hold the severity of the effect of wet- and dry-bulb temperature on mortality and the demographic characteristics of the country fixed, then change the levels of experienced wet- and dry-bulb temperatures. This method facilitates comparison between the effect of current and future climates.

For the climate projections, and again motivated by the need to capture fine scale spatial variability in dry and humid heat, we select the NASA Earth exchange Global Daily Downscaled Projections (NEX-GDDP) dataset, a product statistically downscaled from Coupled Model Intercomparison Project Phase 6 (CMIP6) General Circulation Models (GCMs), as the underlying data for our projections. This downscaled product provides daily data at a spatial resolution of 0.25 de-

grees, significantly more fine than the underlying CMIP6 models which primarily have a resolution of approximately 1 degree. In order to apply the statistical model to various future periods and measure the potential changes in the differential impacts of humid and dry heat on human health, the variables necessary for projections include daily mean dry-bulb temperature, daily mean specific humidity, daily mean pressure, and daily total precipitation. Because the NEX-GDDP dataset does not supply daily mean pressure data, we use an elevation-based approximation at each point. While this method ignores the wet-bulb temperature effects of temporal pressure fluctuations, the resultant bias is no more than approximately 0.25°C [83]. Twenty six models are selected based on their inclusion of these variables. We perform projections for one ensemble member using greenhouse gas emission pathways from four Shared Socioeconomic Pathway (SSP) scenarios, namely SSPs 1-2.6, 2-4.5, 3-7.0, and 5-8.5.

We then use percentile mapping to generate synthetic time series for each meteorological variable (dry-bulb temperature and precipitation as downloaded directly from NEX-GDDP; wet-bulb temperature calculated from NEX-GDDP data as described above) during an end-of-century period. Within each model and SSP GHG emissions scenario, data from the historical and future periods are binned into 1 percentile bins (e.g., 1st percentile, 2nd percentile, ..., 99th percentile). The delta change for each variable in these percentiles is computed between the historical period and future periods. These percentile-specific change factors are then applied to the corresponding percentile days in the observational station data. This approach retains any seasonality and internal variability recorded in the observational historical period, is flexible, and allows for the mean and higher moments of the future distribution of temperatures to be different than what was observed historically.

To ensure comparability between average annual outcomes in the past and in projections, we calculate past average annual outcomes using only exposures from 1998–2014. This period represents the overlap between our mortality data (1998–2019) and the historical period for NEX-GDDP models (1950–2015). The mid-century (2043–2059) and end-of-century (2083–2099) periods are defined to be the same length as this past period, 17 years. Throughout, we use the term "historical" to refer to the period 1998–2014; when discussing the data period over which we resolve our

exposure–response functions (1998–2019), we use the term “sample period.”

A.4 Additional Figures

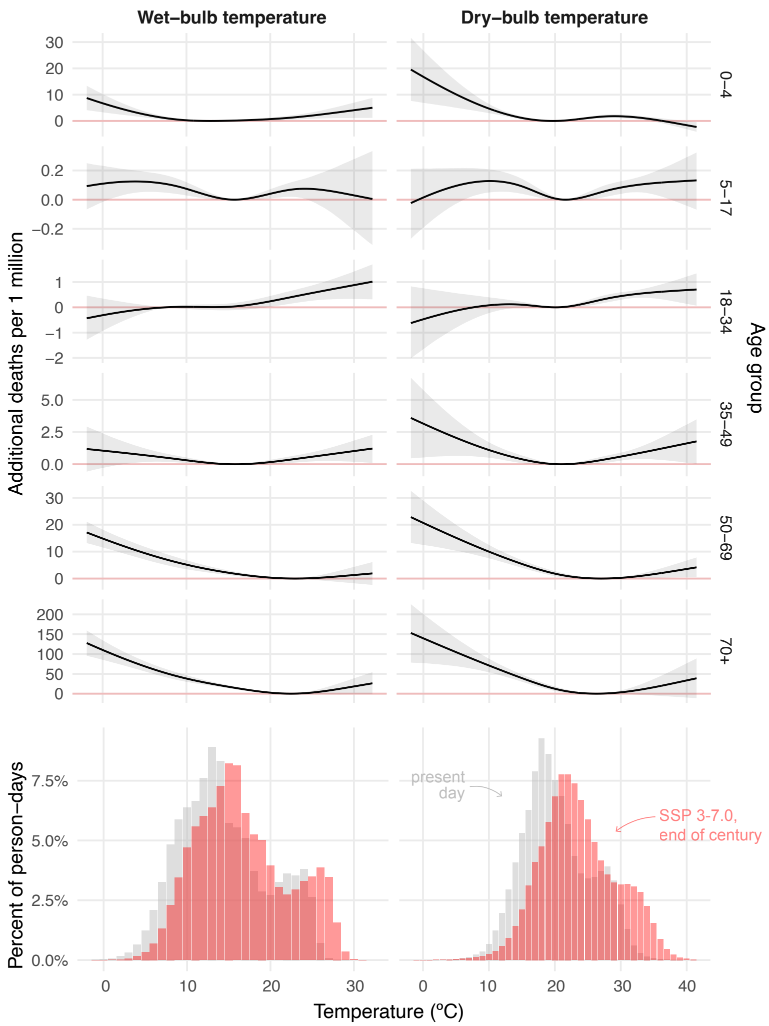

Figure S1: Relationships between mortality and exposure to wet- and dry-bulb temperature by age group in Mexico This figure mirrors Figure 1, but expresses outcomes as absolute changes in deaths and adds a column for dry-bulb temperature. The top panels show the additional effects of 1 million person-days of exposure to the indicated daily average wet- and dry-bulb temperatures (x-axis) on mortality (y-axis); exposure and mortality are in terms of the indicated age group. Bands around each function indicate 95% confidence intervals. The bottom panel shows the distribution of daily average wet- and dry-bulb temperatures in Mexico throughout our sample period as well as the ensemble mean of projected temperatures under the SSP 3-7.0 emission scenario at the end of the century (2083–2099); we impose no change in population distribution or size.

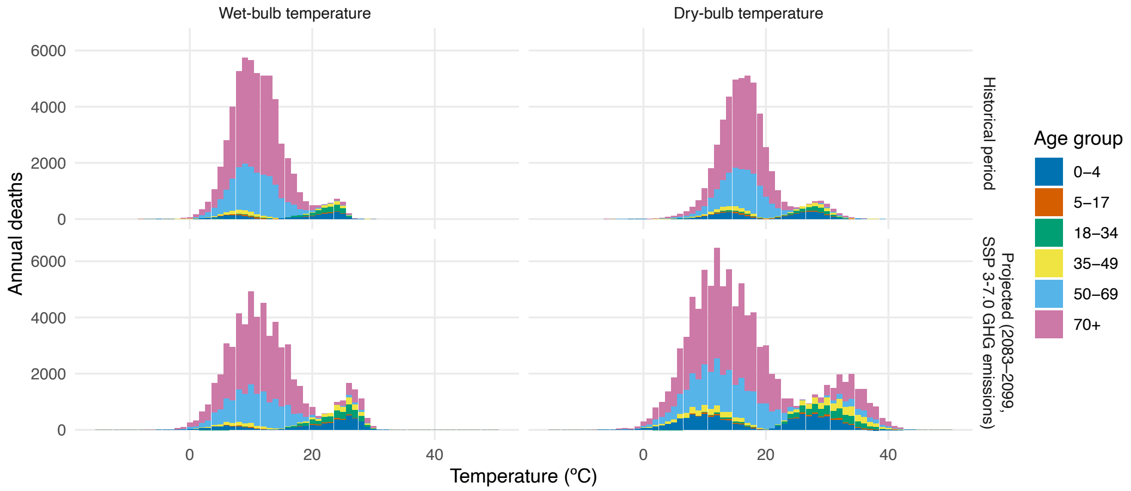

Figure S2: Historical and projected annual temperature-related deaths in Mexico for degree bins of wet- and dry-bulb temperature
This figure mirrors Figure 2, but adds a column for dry-bulb temperature. The panels show average annual temperature-related deaths resulting from exposure to days with the average wet- or dry-bulb temperatures shown on the x-axis during the historical period (top panels) and at the end of the century (2083–2099) under the SSP 3-7.0 emission scenario (bottom panels) across six age groups in Mexico. Projections assume that demographics, socioeconomic characteristics, and the population distribution remain fixed at their historical values. The figure shows mean projected deaths; see Figure S3 for projections with uncertainty.

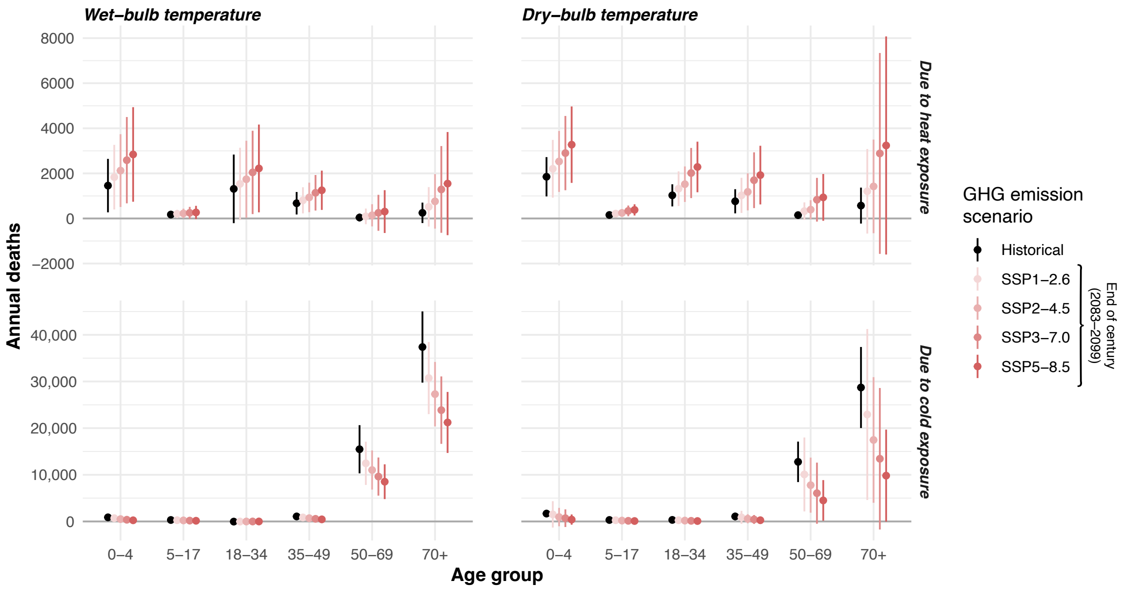

Figure S3: Historical and projected annual deaths due to heat and cold exposure by age group for wet- and dry-bulb temperature

This figure mirrors Figure 3, but adds a column for dry-bulb temperature. The figure depicts the annual number of deaths attributed to heat and cold exposure in Mexico historically and under temperatures prevailing at the end of the century in four greenhouse gas emission scenarios. Top panels indicate values for heat exposure, whereas bottom panels indicate values for cold exposure. Estimates using wet-bulb temperature are shown in the left panels, whereas those derived using dry-bulb temperature are shown in the right panels. Whiskers above and below each estimate depict 95% confidence intervals net of both econometric and climate uncertainty. Note that the range of the y-axis in the bottom panels is roughly five times the range of the y-axis in the top panels.

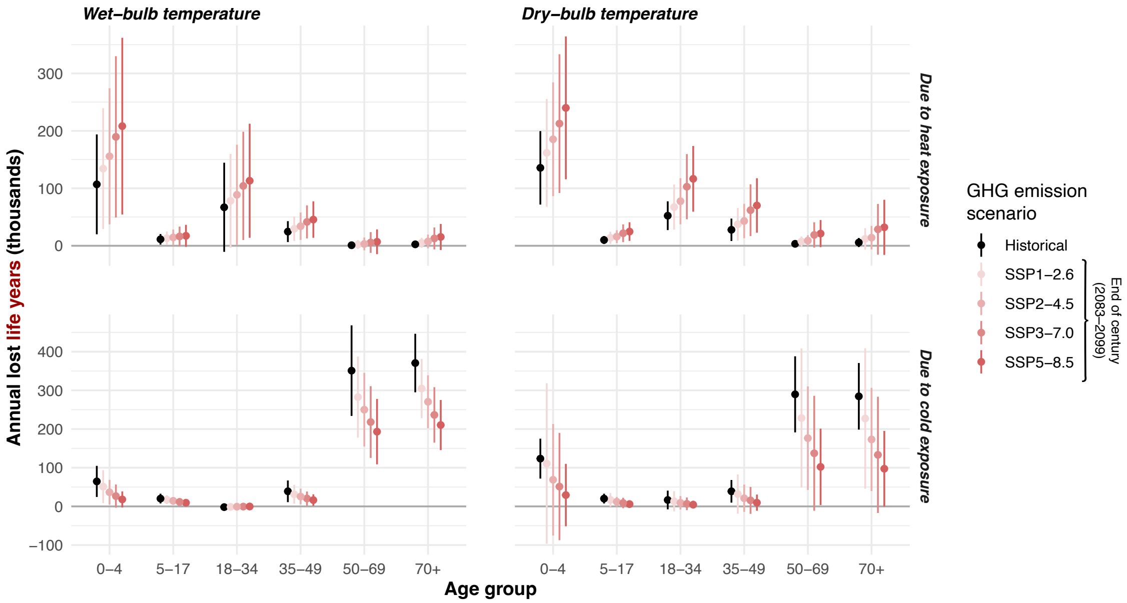

Figure S4: Historical and projected annual lost life years due to heat and cold exposure by age group for wet- and dry-bulb temperature

This figure mirrors Figure 4, but with the outcome as lost life years, rather than deaths. Potential remaining life years are taken from the UN World Population Prospects 2022, and are aggregated to time-invariant age group values by taking a population-weighted average across single age bins and years. The figure depicts the annual number of lost life years attributed to heat and cold exposure in Mexico historically and under temperatures prevailing at the end of the century in four greenhouse gas emission scenarios. Top panels indicate values for heat exposure, whereas bottom panels indicate values for cold exposure. Estimates using wet-bulb temperature are shown in the left panels, whereas those derived using dry-bulb temperature are shown in the right panels. Whiskers above and below each estimate depict 95% confidence intervals net of both econometric and climate uncertainty.

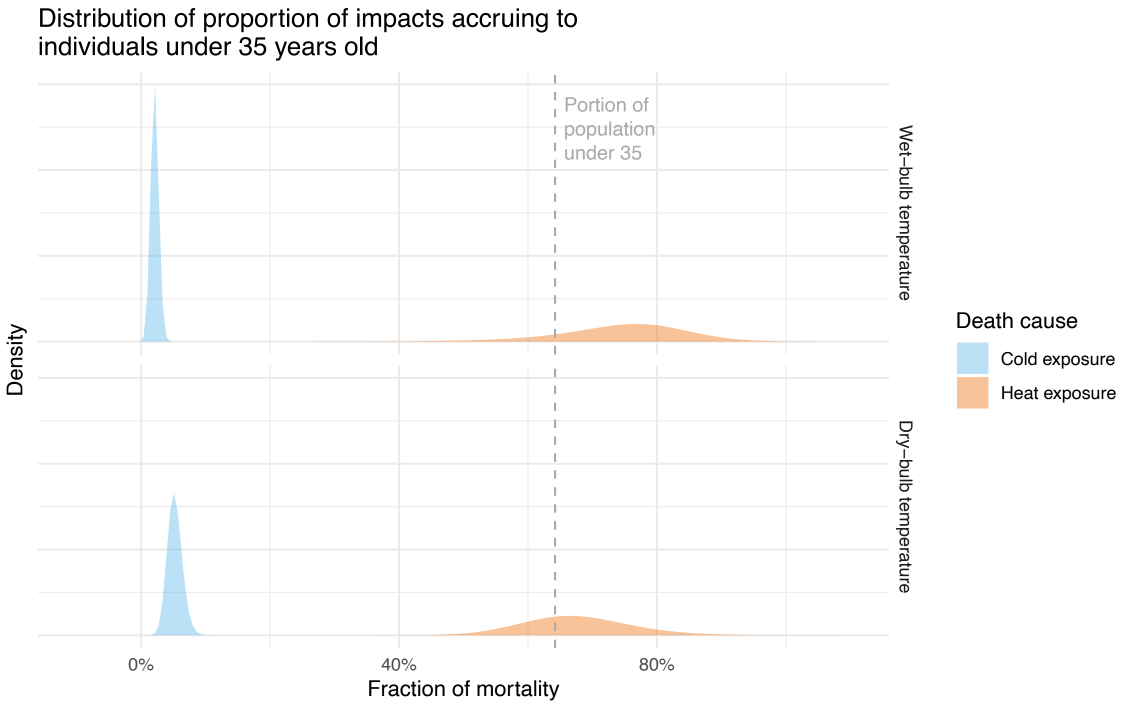

Figure S5: Distribution of estimates of the historical proportion of heat and cold-related deaths occurring to individuals under 35 years old.

Distributions depict the density of bootstrap samples i of $\frac{\text{deaths\_under\_35}_{i,\text{type}}}{\text{all\_deaths}_{i,\text{type}}}$, where the number of samples is 10,000 and type $\in$ {cold-related, heat-related}. Results using wet-bulb temperature are shown in the top panel, whereas results using dry-bulb temperature are shown in the bottom panel. As of the 2010 Census, around 63% of the Mexican population is under 35, a value labeled on the plot with a dashed vertical line.

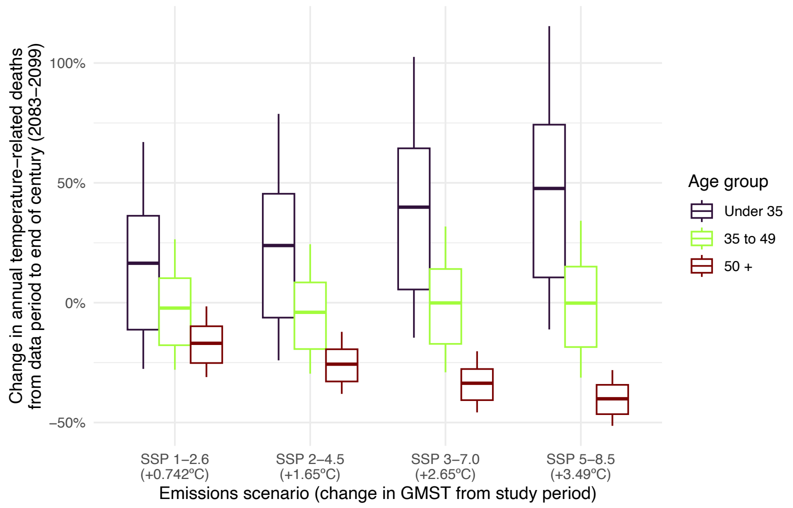

Figure S6: Projected percent change in annual deaths in Mexico under different climate change scenarios using wet-bulb temperature projections

The figure shows the percent change in average annual deaths by end-of-century (2083–2099) relative to the historical period for four different climate scenarios, indicated on the x-axis, and for three different age groups: those under 35 years old, between 35 and 49 years old, and over 50 years old. Box boundaries depict the 25th and 75th percentile of bootstrap estimates, while whiskers depict the 10th and 90th percentiles. Projections assume that demographics, socioeconomic characteristics, and the population distribution remain fixed at their historical values. The differences in levels is shown in Figure S7.

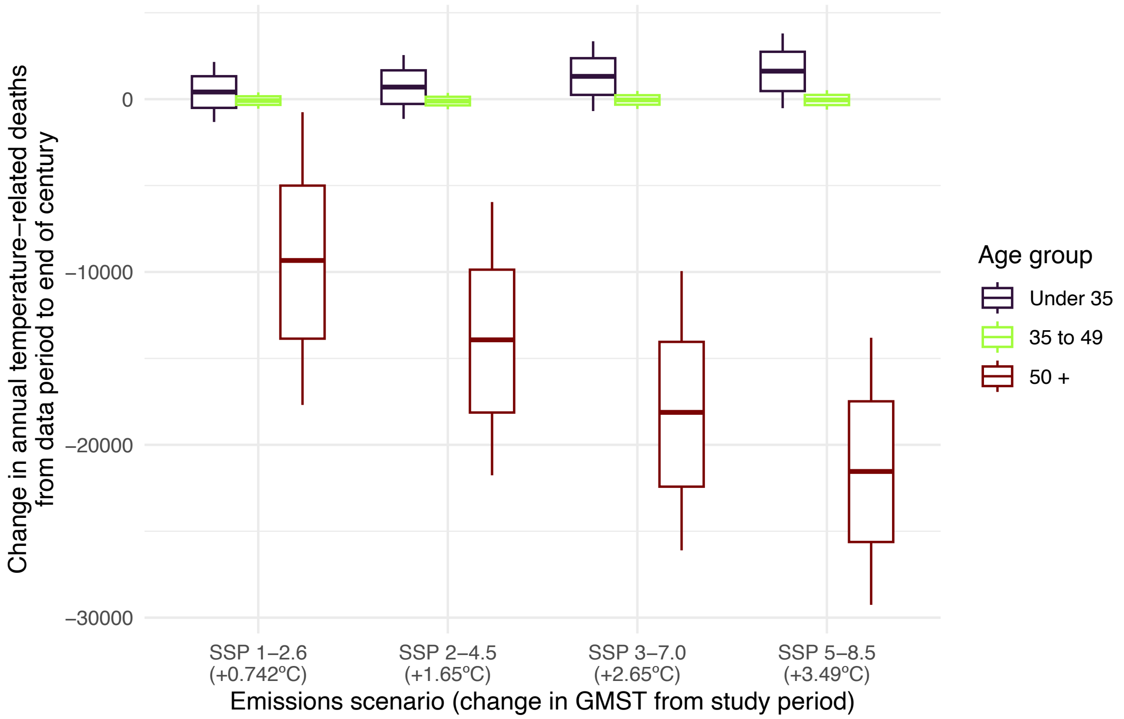

Figure S7: Projected change in annual deaths in Mexico under different climate change scenarios using wet-bulb temperature projections

The figure shows the level of average annual deaths by end-of-century (2083–2099) relative to the historical period for four different climate scenarios, indicated on the x-axis, for three different age groups: those under 35 years old, those between 35 and 49 years old, and those over 50 years old. Box boundaries depict the 25th and 75th percentile of bootstrap estimates, while whiskers depict the 10th and 90th percentiles. Projections assume that demographics, socioeconomic characteristics, and the population distribution remain fixed at their historical values. Figure S6 shows the same information but in terms of percent change.

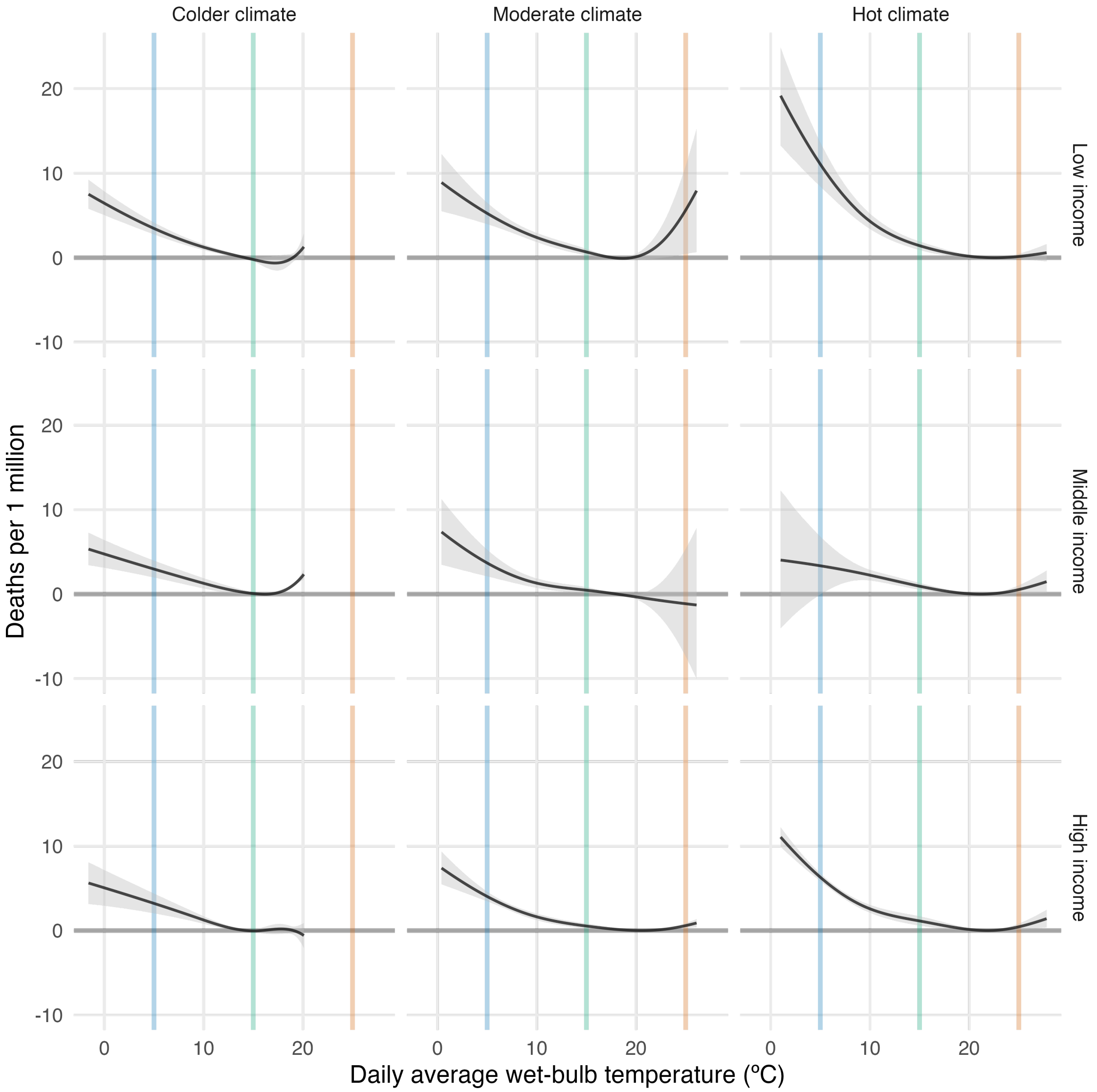

Figure S8: Heterogeneity in effects by climate and income tercile
Relationship between average daily wet–bulb temperature and additional deaths per 1 million person–days of exposure by income and climate tercile. Income terciles divide Mexico's municipalities into three roughly equal-population groups by their average earned income as reported by the Mexican census; climate terciles divide Mexico's municipalities into three roughly equal-population groups by their average annual wet-bulb temperature during the data period. Effects are for the overall population (pooled across age groups). Shaded area represents a confidence interval of 95%. Dose–response functions are shown to each tercile's population-weighted 0.01 and 99.99 percentile temperature exposure (colored vertical lines are added at 5, 15, and 25°C to aid visual comparison).

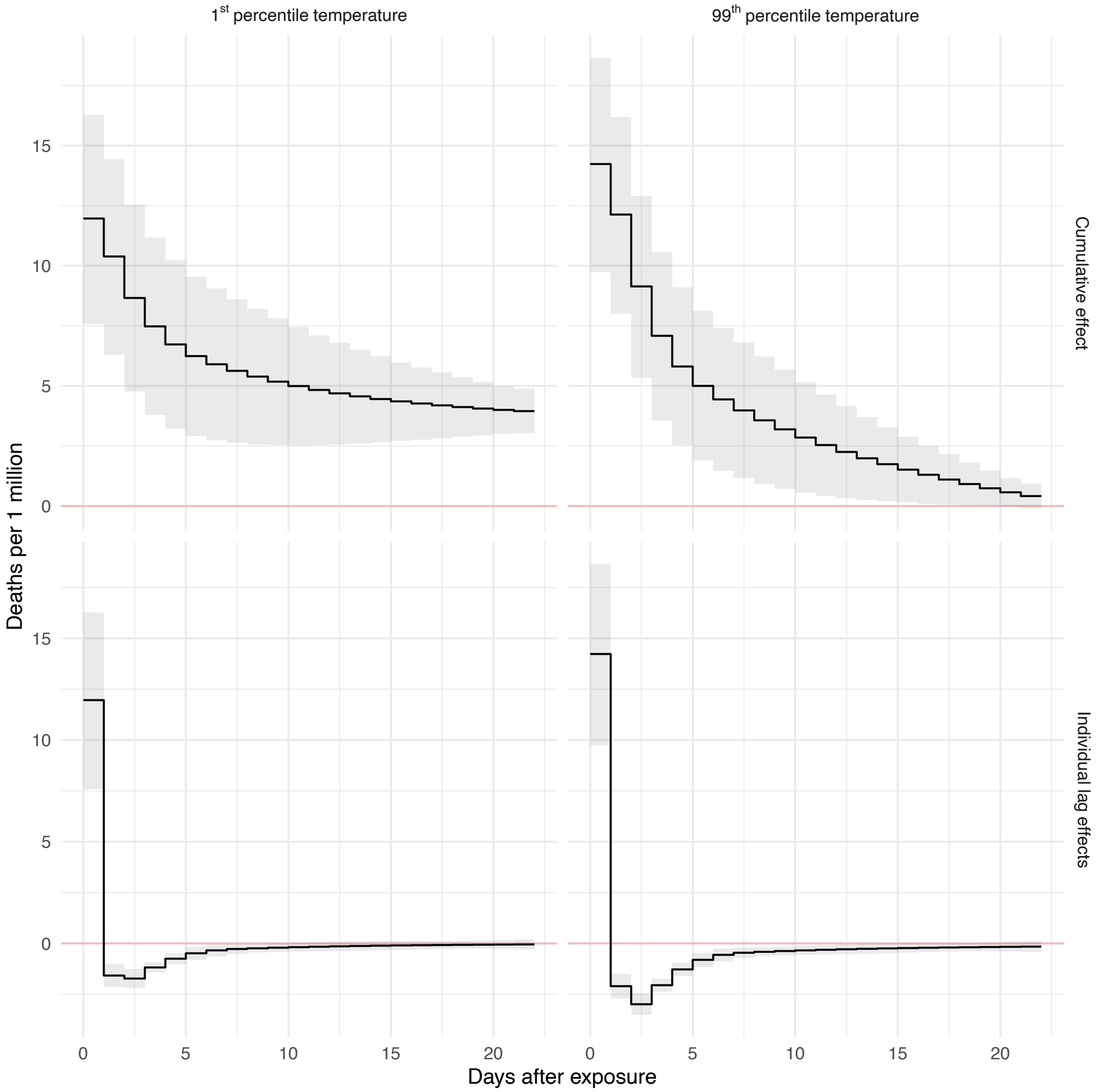

Figure S9: Lag–response relationship when estimating Eq. S-1
Additional deaths per 1 million person–days of exposure to average daily 1st- (left panels) and 99th- (right panels) percentile wet-bulb temperatures (approximately 3.96 and 25.25°C, respectively). Effects are for the overall population (pooled across age groups). Top panels show cumulative effects across the 21-day lag period; bottom panels show the effects at individual lags. Shaded area represents a confidence interval of 95%.

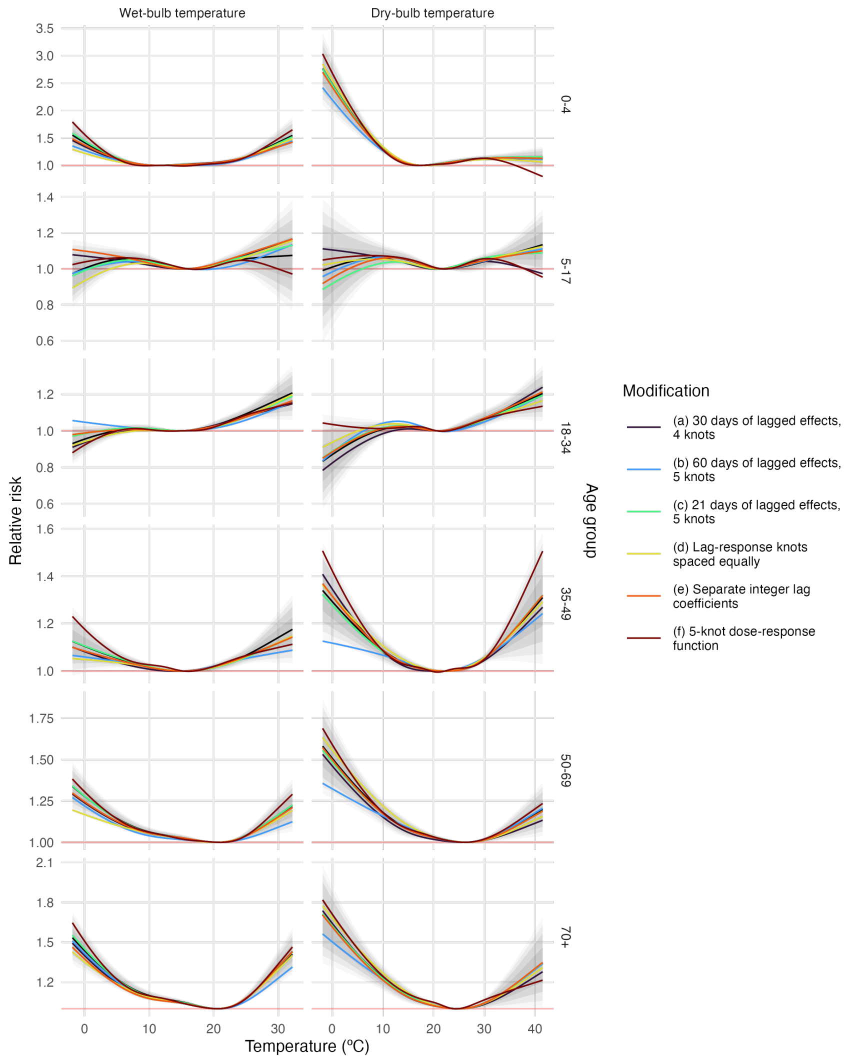

Figure S10: Robustness of results to modifications to Eq. S-1
Estimated relationship between wet- and dry-bulb temperature and relative risk of mortality by age group for alternate model specifications. (a) extends the main model's 21-day lag period to 30 days and adds a single knot to the 3-knot lag–response specification to preserve a similar degree of flexibility in the lag–response function; (b) extends this further to 60 days and adds another knot to the lag–response function. (c) adds two additional knots to the main model's 21-day lag structure, bringing the total number of lag–response function knots to five. (d) changes the placement of the main model's three lag–response knots, placing them equally throughout the lag space instead of spaced logarithmically. (e) includes separate coefficients for each integer lag in the main model's 21-day lag space. (f) changes the specification of the dose–response function, increasing the number of knots from three to five, with placement following [81]. Shaded bands around the main model (black line) result indicate 95, 90, 80, and 50% confidence intervals.

Table S1: Additional deaths per 1 million person–days of exposure to an average daily wet-bulb temperature in the indicated bin. Values were determined by estimating Equation S-1, but with a discretized version of the nonlinear dose–response component of $f_a(\cdot)$. Standard errors are shown in parentheses.

	**Age group**					
**Temperature (°C)**	0–4	5–17	18–34	35–49	50–69	70+
<3	2.923<br>(0.91)	0.057<br>(0.101)	-0.661<br>(0.178)	0.315<br>(0.385)	5.366<br>(1.255)	49.42<br>(9.21)
[3,6)	1.582<br>(0.504)	0.051<br>(0.041)	0.142<br>(0.077)	0.851<br>(0.194)	4.466<br>(0.83)	32.834<br>(6.13)
[6,9)	0.334<br>(0.327)	0.063<br>(0.033)	0.029<br>(0.044)	0.165<br>(0.134)	2.352<br>(0.581)	18.623<br>(3.643)
[9,12)	0.124<br>(0.218)	0.041<br>(0.026)	0.042<br>(0.037)	0.217<br>(0.101)	1.546<br>(0.37)	10.819<br>(2.361)
[12,15)	0.222<br>(0.216)	0.015<br>(0.027)	0<br>(0)	0.059<br>(0.088)	0.932<br>(0.369)	8.927<br>(2.212)
[15,18)	0<br>(0)	0<br>(0)	0.032<br>(0.053)	0<br>(0)	0.449<br>(0.286)	1.946<br>(1.353)
[18,21)	0.444<br>(0.208)	0.024<br>(0.022)	0.069<br>(0.067)	0.086<br>(0.09)	0<br>(0)	0<br>(0)
[21,24)	0.648<br>(0.22)	0.048<br>(0.024)	0.218<br>(0.071)	0.331<br>(0.114)	0.114<br>(0.227)	0.804<br>(1.062)
[24,27)	1.441<br>(0.307)	0.058<br>(0.045)	0.303<br>(0.083)	0.483<br>(0.148)	1.174<br>(0.488)	9.606<br>(2.47)
27+	3.392<br>(0.982)	0.008<br>(0.35)	1.049<br>(1.158)	2.061<br>(1.272)	3.575<br>(2.399)	41.039<br>(8.131)

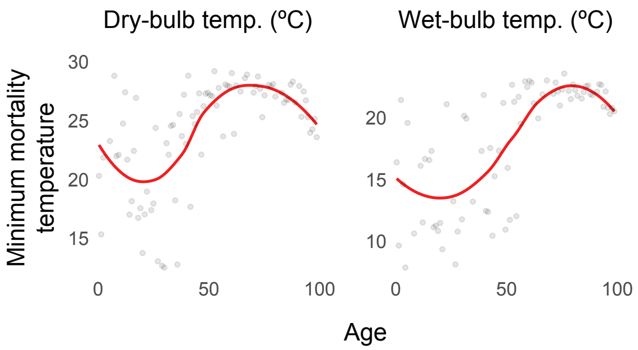

Figure S11: Minimum mortality temperatures across age groups in Mexico
This figure demonstrates the relationship between age and the temperature at which mortality is minimized ("MMT"). A locally-weighted regression line is added to aid in visual inspection, as these parameter estimates are subject to noise. These MMTs are determined by separately estimating Eq. S-1 for each age and temperature metric and determining the temperature at which mortality risk is minimized. MMT decreases from birth to the mid-20s, and then increases substantially with age to around age 70 before flattening and decreasing slightly to age 100. Individuals in their mid-20s have a dry-bulb MMT of 20°C and a wet-bulb MMT of 13°C. Dry-bulb MMT peaks at 28°C at age 70 and wet-bulb MMT peaks at 22°C at age 75.

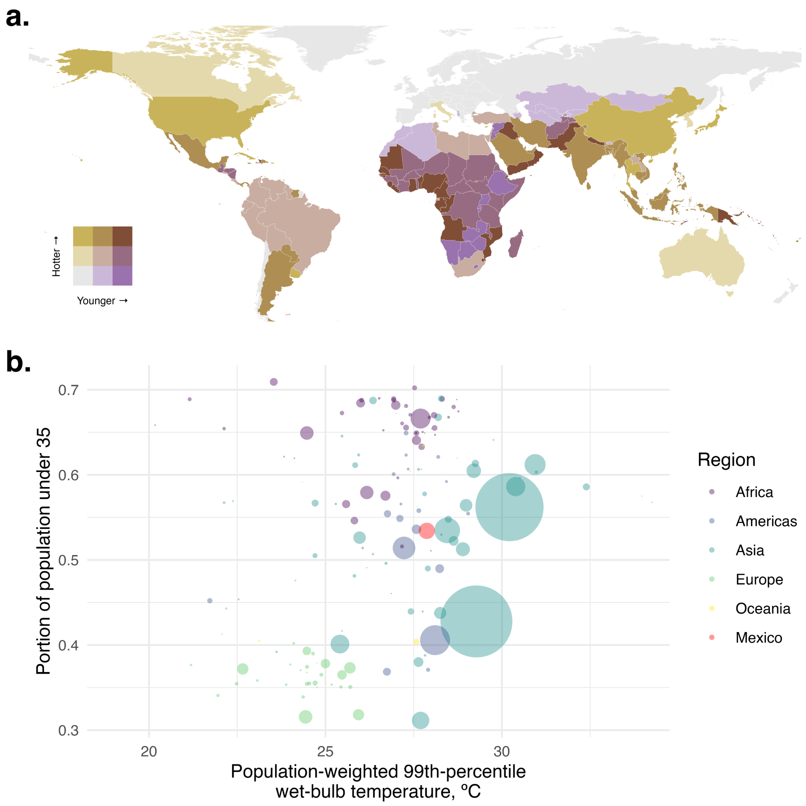

Figure S12: Historical extreme wet-bulb temperature exposures and current portion of population under 35

a. Countries colored according to their tercile of the global distribution of (1) historical extreme wet-bulb temperature exposures: 99th percentile of population-weighted exposures, with temperatures estimates by [32] using ERA-5 Interim values from 1979 to 2017 and population distribution information from [84] and (2) portion of 2010 population under 35 [70]. **b.** Scatterplot of country-level historical extreme wet-bulb temperature exposure and the portion of the 2010 population under 35. Points are scaled according to total population size.

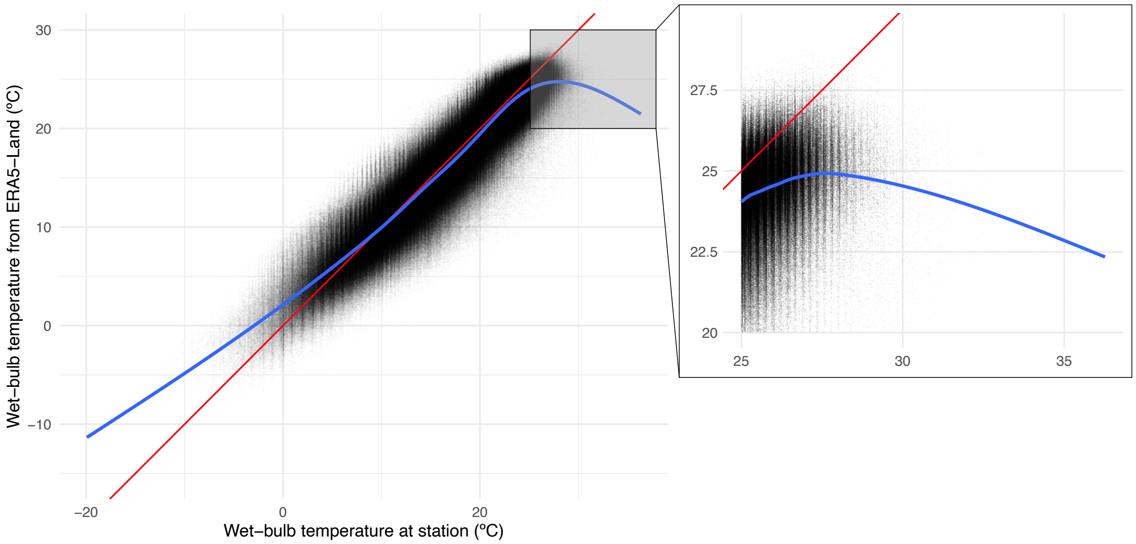

Figure S13: Daily average wet-bulb temperatures recorded at stations and in ERA5-Land

Data spans the period 1998–2019. Each point represents a pair of observations at a weather station (mean of sub-daily values) in Mexico and the corresponding value of ERA5-Land at that position and time (mean of hourly values). The blue line is a generalized additive model of cubic regression splines estimated by REML. The red line, for comparison, illustrates what would be observed with a one-to-one correspondence.

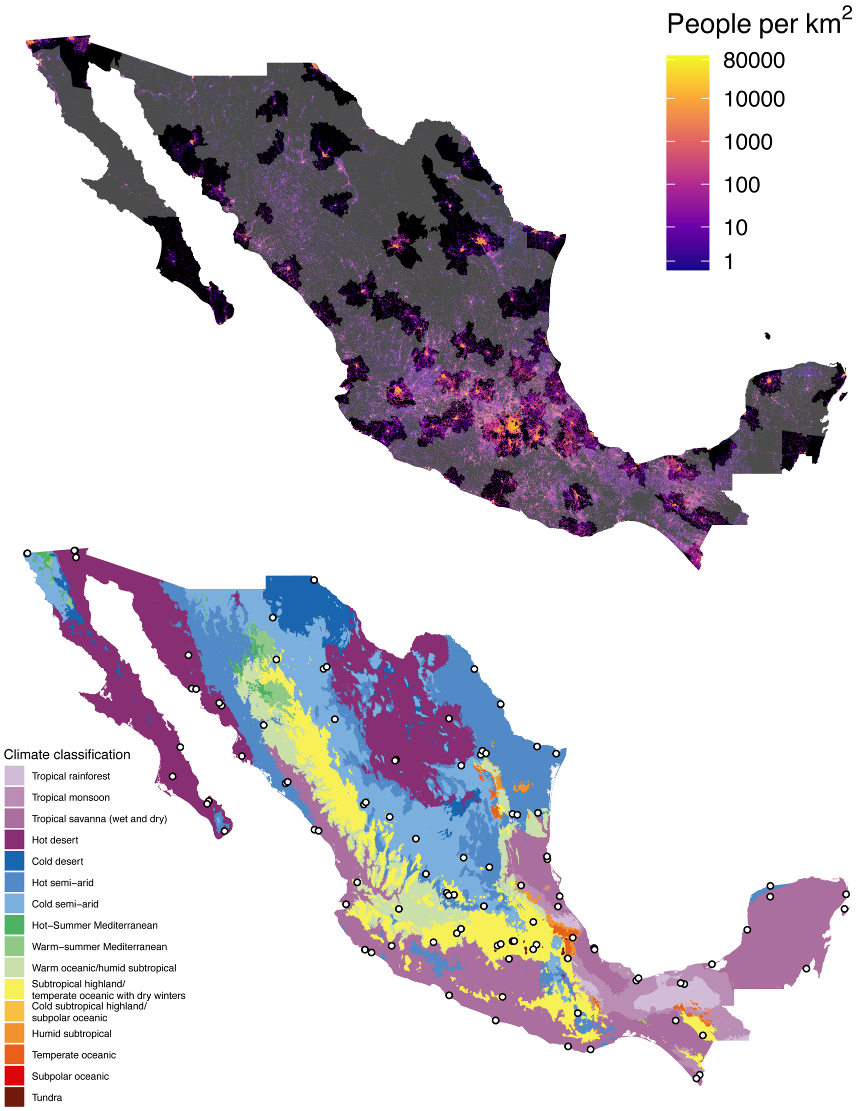

Figure S14: Map of population, municipalities, weather monitor locations, and climate zones in Mexico
The top panel of this figure shows the population density of Mexico (background color gradient) in the municipalities analyzed. The subset of municipalities dropped from our analysis (because they are too far from weather stations) are grayed out. The bottom panel of this figure shows the location of weather stations used in our analysis (white points with black outlines) and Köppen climate zones (background color gradient).

REFERENCES AND NOTES

1. A. Gasparrini, Y. Guo, M. Hashizume, E. Lavigne, A. Zanobetti, J. Schwartz, A. Tobias, S. Tong, J. Rocklöv, B. Forsberg, M. Leone, M. de Sario, M. L. Bell, Y.-L. L. Guo, C.-F. Wu, H. Kan, S.-M. Yi, M. de Sousa Zanotti Stagliorio Coelho, P. H. N. Saldiva, Y. Honda, H. Kim, B. Armstrong, Mortality risk attributable to high and low ambient temperature: A multicountry observational study. *Lancet* **386**, 369–375 (2015).

2. D. Mitchell, C. Heaviside, S. Vardoulakis, C. Huntingford, G. Masato, B. P. Guillod, P. Frumhoff, A. Bowery, D. Wallom, M. Allen, Attributing human mortality during extreme heat waves to anthropogenic climate change. *Environ. Res. Lett.* **11**, 074006 (2016).

3. A. M. Vicedo-Cabrera, N. Scovronick, F. Sera, D. Royé, R. Schneider, A. Tobias, C. Astrom, Y. Guo, Y. Honda, D. M. Hondula, R. Abrutzky, S. Tong, M. S. Z. S. Coelho, P. H. N. Saldiva, E. Lavigne, P. M. Correa, N. V. Ortega, H. Kan, S. Osorio, J. Kyselý, A. Urban, H. Orru, E. Indermitte, J. J. K. Jaakkola, N. Ryti, M. Pascal, A. Schneider, K. Katsouyanni, E. Samoli, F. Mayvaneh, A. Entezari, P. Goodman, A. Zeka, P. Michelozzi, F. de'Donato, M. Hashizume, B. Alahmad, M. H. Diaz, C. D. L. C. Valencia, A. Overcenco, D. Houthuijs, C. Ameling, S. Rao, F. di Ruscio, G. Carrasco-Escobar, X. Seposo, S. Silva, J. Madureira, I. H. Holobaca, S. Fratianni, F. Acquaotta, H. Kim, W. Lee, C. Iniguez, B. Forsberg, M. S. Ragettli, Y. L. L. Guo, B. Y. Chen, S. Li, B. Armstrong, A. Aleman, A. Zanobetti, J. Schwartz, T. N. Dang, D. V. Dung, N. Gillett, A. Haines, M. Mengel, V. Huber, A. Gasparrini, The burden of heat-related mortality attributable to recent human-induced climate change. *Nat. Clim. Chang.* **11**, 492–500 (2021).

4. R. D. Bressler, The mortality cost of carbon. *Nat. Commun.* **12**, 4467 (2021).

5. R. D. Bressler, F. C. Moore, K. Rennert, D. Anthoff, Estimates of country level temperature-related mortality damage functions. *Sci. Rep.* **11**, 20282 (2021).

6. T. Carleton, A. Jina, M. Delgado, M. Greenstone, T. Houser, S. Hsiang, A. Hultgren, R. E. Kopp, K. E. McCusker, I. Nath, J. Rising, A. Rode, H. K. Seo, A. Viaene, J. Yuan, A. T. Zhang, Valuing the global mortality consequences of climate change accounting for adaptation costs and benefits. *Q. J. Econ.* **69**, 2037–2105 (2022).

7. K. Chen, R. M. Horton, D. A. Bader, C. Lesk, L. Jiang, B. Jones, L. Zhou, X. Chen, J. Bi, P. L. Kinney, Impact of climate change on heat-related mortality in Jiangsu Province, China. *Environ. Pollut.* **224**, 317–325 (2017).

8. K. R. Cromar, S. C. Anenberg, J. R. Balmes, A. A. Fawcett, M. Ghazipura, J. M. Gohlke, M. Hashizume, P. Howard, E. Lavigne, K. Levy, J. Madrigano, J. A. Martinich, E. A. Mordecai, M. B. Rice, S. Saha, N. C. Scovronick, F. Sekercioglu, E. R. Svendsen, B. F. Zaitchik, G. Ewart, Global health impacts for economic models of climate change: A systematic review and meta-analysis. *Ann. Am. Thorac. Soc.* **19**, 1203–1212 (2022).

9. O. Deschênes, M. Greenstone, Climate change, mortality, and adaptation: Evidence from annual fluctuations in weather in the US. *Am. Econ. J. Appl. Econ.* **3**, 152–185 (2011).

10. A. Gasparrini, Y. Guo, F. Sera, A. M. Vicedo-Cabrera, V. Huber, S. Tong, M. de Sousa Zanotti Stagliorio Coelho, P. H. Nascimento Saldiva, E. Lavigne, P. Matus Correa, N. Valdes Ortega, H. Kan, S. Osorio, J. Kyselý, A. Urban, J. J. K. Jaakkola, N. R. I. Ryti, M. Pascal, P. G. Goodman, A. Zeka, P. Michelozzi, M. Scortichini, M. Hashizume, Y. Honda, M. Hurtado-Diaz, J. Cesar Cruz, X. Seposo, H. Kim, A. Tobias, C. Iñiguez, B. Forsberg, D. O. Åström, M. S. Ragettli, Y. L. Guo, C.F. Wu, A. Zanobetti, J. Schwartz, M. L. Bell, T. N. Dang, D. D. van, C. Heaviside, S. Vardoulakis, S. Hajat, A. Haines, B. Armstrong, Projections of temperature-related excess mortality under climate change scenarios. *Lancet Planetary Health* **1**, E360–E367 (2017).

11. S. Hajat, S. Vardoulakis, C. Heaviside, B. Eggen, Climate change effects on human health: Projections of temperature-related mortality for the UK during the 2020s, 2050s and 2080s. *J. Epidemiol. Community Health* **68**, 641–648 (2014).

12. S. Hales, S. Kovats, S. Lloyd, D. Campbell-Lendrum, *Quantitative Risk Assessment of the Effects of Climate Change on Selected Causes of Death, 2030s and 2050s* (World Health Organization, 2014).

13. Y. Honda, M. Kondo, G. McGregor, H. Kim, Y.-L. Guo, Y. Hijioka, M. Yoshikawa, K. Oka, S. Takano, S. Hales, R. S. Kovats, Heat-related mortality risk model for climate change impact projection. *Environ. Health Prev. Med.* **19**, 56–63 (2014).

14. T. Houser, S. Hsiang, R. Kopp, K. Larsen, M. Delgado, A. Jina, M. Mastrandrea, S. Mohan, R. Muir-Wood, D. J. Rasmussen, J. Rising, P. Wilson, *Economic Risks of Climate Change: An American Prospectus* (Columbia Univ. Press, 2015).

15. S. L. Kingsley, M. N. Eliot, J. Gold, R. R. Vanderslice, G. A. Wellenius, Current and projected heat-related morbidity and mortality in Rhode Island. *Environ. Health Perspect.* **124**, 460–467 (2016).

16. K. Knowlton, B. Lynn, R. A. Goldberg, C. Rosenzweig, C. Hogrefe, J. K. Rosenthal, P. L. Kinney, Projecting heat-related mortality impacts under a changing climate in the New York City region. *Am. J. Public Health* **97**, 2028–2034 (2007)

17. D.-W. Kim, R. C. Deo, J.-H. Chung, J.-S. Lee, Projection of heat wave mortality related to climate change in Korea. *Nat. Hazards* **80**, 623–637 (2016).

18. J. Y. Lee, H. Kim, Projection of future temperature-related mortality due to climate and demographic changes. *Environ. Int.* **94**, 489–494 (2016).

19. T. Li, R. M. Horton, P. L. Kinney, Projections of seasonal patterns in temperature- related deaths for Manhattan, New York. *Nat. Clim. Chang.* **3**, 717–721 (2013)

20. A. Marsha, S. R. Sain, M. J. Heaton, A. J. Monaghan, O. V. Wilhelmi, Influences of climatic and population changes on heat-related mortality in Houston, Texas, USA. *Clim. Change* **146**, 471–485 (2018)

21. È. Martínez-Solanas, M. Quijal-Zamorano, H. Achebak, D. Petrova, J.-M. Robine, F. R. Herrmann, X. Rodó, J. Ballester, Projections of temperature-attributable mortality in Europe: A time series analysis of 147 contiguous regions in 16 countries. *Lancet. Planetary Health* **5**, E446–E454 (2021).

22. R. D. Peng, J. F. Bobb, C. Tebaldi, L. M. Daniel, M. L. Bell, F. Dominici, Toward a quantitative estimate of future heat wave mortality under global climate change. *Environ. Health Perspect.* **119**, 701–706 (2011).

23. E. Petkova, R. Horton, D. Bader, P. Kinney, Projected heat-related mortality in the U.S. urban Northeast. *Int. J. Environ. Res. Public Health* **10**,6734–6747 (2013).

24. J. D. Schwartz, M. Lee, P. L. Kinney, S. Yang, D. Mills, M. C. Sarofim, R. Jones, R. Streeter, A. S. Juliana, J. Peers, R. M. Horton, Projections of temperature-attributable premature deaths in 209 U.S. cities using a cluster-based Poisson approach. *Environ. Health* **14**, 85 (2015).

25. D. Shindell, Y. Zhang, M. Scott, M. Ru, K. Stark, K. L. Ebi, The effects of heat exposure on human mortality throughout the United States. *GeoHealth* **4**, e2019GH000234 (2020).

26. J. Yang, M. Zhou, Z. Ren, M. Li, B. Wang, D. L. Liu, C.-Q. Ou, P. Yin, J. Sun, S. Tong, H. Wang, C. Zhang, J. Wang, Y. Guo, Q. Liu, Projecting heat-related excess mortality under climate change scenarios in China. *Nat. Commun.* **12**, 1039 (2021)

27. B. Zhang, G. Li, Y. Ma, X. Pan, Projection of temperature-related mortality due to cardiovascular disease in beijing under different climate change, population, and adaptation scenarios. *Environ. Res.* **162**, 152–159 (2018).

28. J. A. Jáuregui Díaz, M. de Jesús Ávila Sánchez, R. T. Cabañas, Cambios en la mortalidad por eventos climáticos extremos en México entre el 2000 y 2015. *Revista de Estudios Latinoamericanos sobre Reducci'on del Riesgo de Desastres REDER* **4**, 80–94 (2020).

29. T. Li, R. M. Horton, D. A. Bader, M. Zhou, X. Liang, J. Ban, Q. Sun, P. L. Kinney, Aging will amplify the heat-related mortality risk under a changing climate: Projection for the elderly in Beijing, China. *Sci. Rep.* **6**, 28161 (2016).

30. C. Mora, B. Dousset, I. R. Caldwell, F. E. Powell, R. C. Geronimo, C. R. Bielecki, C. W. W. Counsell, B. S. Dietrich, E. T. Johnston, L. V. Louis, M. P. Lucas, M. M. McKenzie, A. G. Shea, H. Tseng, T. W. Giambelluca, L. R. Leon, E. Hawkins, C. Trauernicht, Global risk of deadly heat. *Nat. Clim. Chang.* **7**, 501–506 (2017).

31. B. Armstrong, F. Sera, A. M. Vicedo-Cabrera, R. Abrutzky, D. O. Åström, M. L. Bell, B.Y. Chen, M. de Sousa Zanotti Stagliorio Coelho, P. M. Correa, T. N. Dang, M. H. Diaz, D. V. Dung, B. Forsberg, P. Goodman, Y.L. L. Guo, Y. Guo, M. Hashizume, Y. Honda, E. Indermitte,

C. Íñiguez, H. Kan, H. Kim, J. Kyselý, E. Lavigne, P. Michelozzi, H. Orru, N. V. Ortega, M. Pascal, M. S. Ragettli, P. H. N. Saldiva, J. Schwartz, M. Scortichini, X. Seposo, A. Tobias, S. Tong, A. Urban, C. de la Cruz Valencia, A. Zanobetti, A. Zeka, A. Gasparrini, The role of humidity in associations of high temperature with mortality:a multicountry, multicity study. *Environ. Health Perspect.* **127**, 097007 (2019).

32. C. Raymond, T. Matthews, R. M. Horton, The emergence of heat and humidity too severe for human tolerance. *Sci. Adv.* **6**, eaaw1838 (2020).

33. S. C. Sherwood, M. Huber, An adaptability limit to climate change due to heat stress. *Proc. Natl. Acad. Sci. U.S.A.* **107**, 9552–9555 (2010).

34. D. J. Vecellio, S. T. Wolf, R. M. Cottle, W. L. Kenney, Evaluating the 35°C wet-bulb temperature adaptability threshold for young, healthy subjects (PSU HEAT Project). *J. Appl. Physiol.* **132**, 340–345 (2022).

35. J. Vanos, G. Guzman-Echavarria, J. W. Baldwin, C. Bongers, K. L. Ebi, O. Jay, A physiological approach for assessing human survivability and liveability to heat in a changing climate. *Nat. Commun.* **14**, 7653 (2023).

36. J. W. Baldwin, T. Benmarhnia, K. L. Ebi, O. Jay, N. J. Lutsko, J. K. Vanos, Humidity's role in heat-related health outcomes: A heated debate. *Environ. Health Perspect.* **131**, 055001 (2023).

37. G. Havenith, D. Fiala, Thermal indices and thermophysiological modeling for heat stress. *Compr. Physiol.* **6**, 255–302 (2011)

38. J. R. Buzan, M. Huber, Moist heat stress on a hotter earth. *Annu. Rev. Earth Planet. Sci.* **48**, 623–655 (2020).

39. K. Parsons, *Human Thermal Environments: The Effects of Hot, Moderate, and Cold Environments on Human Health, Comfort, and Performance* (CRC Press, 2014).

40. R. G. Steadman, The assessment of sultriness. Part I: A temperature-humidity index based on human physiology and clothing science. *J. Appl. Meteorol. Climatol.* **18**, 861–873 (1979).

41. K. L. Ebi, A. Capon, P. Berry, C. Broderick, R. de Dear, G. Havenith, Y. Honda, R. S. Kovats, W. Ma, A. Malik, N. B. Morris, L. Nybo, S. I. Seneviratne, J. Vanos, O. Jay, Hot weather and heat extremes: Health risks. *Lancet* **398**, 698–708 (2021).

42. E. Gallo, M. Quijal-Zamorano, R. F. Méndez Turrubiates, C. Tonne, X. Basagaña, H. Achebak, J. Ballester, Heat-related mortality in Europe during 2023 and the role of adaptation in protecting health. *Nat. Med.* 10.1038/s41591-024-03186-1 (2024).

43. R. Davies-Jones, An efficient and accurate method for computing the wet-bulb temperature along pseudoadiabats. *Mon. Weather Rev.* **136**, 2764–2785 (2008).

44. J. Schwartz, Harvesting and long term exposure effects in the relation between air pollution and mortality. *Am. J. Epidemiol.* **151**, 440–448 (2000).

45. B. Thrasher, E. P. Maurer, C. McKellar, P. B. Duffy, Bias correcting climate model simulated daily temperature extremes with quantile mapping. *Hydrol. Earth Syst. Sci.* **16**, 3309–3314 (2012).

46. K. Riahi, D. P. van Vuuren, E. Kriegler, J. Edmonds, B. C. O'Neill, S. Fujimori, N. Bauer, K. Calvin, R. Dellink, O. Fricko, W. Lutz, A. Popp, J. C. Cuaresma, S. KC, M. Leimbach, L. Jiang, T. Kram, S. Rao, J. Emmerling, K. Ebi, T. Hasegawa, P. Havlik, F. Humpenöder, L. A. da Silva, S. Smith, E. Stehfest, V. Bosetti, J. Eom, D. Gernaat, T. Masui, J. Rogelj, J. Strefler, L. Drouet, V. Krey, G. Luderer, M. Harmsen, K. Takahashi, L. Baumstark, J. C. Doelman, M. Kainuma, Z. Klimont, G. Marangoni, H. Lotze-Campen, M. Obersteiner, A. Tabeau, M. Tavoni, The Shared Socioeconomic Pathways and their energy, land use, and greenhouse gas emissions implications: An overview. *Glob. Environ. Change* **42**, 153–168 (2017).

47. R. J. H. Dunn, K. M. Willett, D. E. Parker, L. Mitchell, Expanding HadISD: Quality controlled, sub-daily station data from 1931. *Geosci. Instrum. Methods Data Syst.* **5**, 473–491 (2016).

48. J. Muñoz-Sabater, E. Dutra, A. Agustí-Panareda, C. Albergel, G. Arduini, G. Balsamo, S. Boussetta, M. Choulga, S. Harrigan, H. Hersbach, B. Martens, D. G. Miralles, M. Piles, N. J.

Rodríguez-Fernández, E. Zsoter, C. Buontempo, J.-N. Thépaut, ERA5-Land: A state-of-the-art global reanalysis dataset for landapplications. *Earth Syst. Sci. Data* **13**, 4349–4383 (2021).

49. C. Raymond, D. Waliser, B. Guan, H. Lee, P. Loikith, E. Massoud, A. Sengupta, D. Singh, A. Wootten, Regional and elevational patterns of extreme heat stress change in the US. *Environ. Res. Lett.* **17**, 064046 (2022).

50. F. Cohen, A. Dechezleprêtre, Mortality, temperature, and public health provision: Evidence from Mexico. *Am. Econ. J. Econ. Policy* **14**, 161–192 (2022).

51. C. M. Powis, D. Byrne, Z. Zobel, K. N. Gassert, A. C. Lute, C. R. Schwalm, Observational and model evidence together support wide-spread exposure to noncompensable heat under continued global warming. *Sci. Adv.* **9**, eadg9297 (2023).

52. Y. Wu, S. Li, Q. Zhao, B. Wen, A. Gasparrini, S. Tong, A. Overcenco, A. Urban, A. Schneider, A. Entezari, A. M. Vicedo-Cabrera, A. Zanobetti, A. Analitis, A. Zeka, A. Tobias, B. Nunes, B. Alahmad, B. Armstrong, B. Forsberg, S.C. Pan, C. Íñiguez, C. Ameling, C. de la Cruz Valencia, C. Åström, D. Houthuijs, D. van Dung, D. Royé, E. Indermitte, E. Lavigne, F. Mayvaneh, F. Acquaotta, F. de'Donato, S. Rao, F. Sera, G. Carrasco-Escobar, H. Kan, H. Orru, H. Kim, I.-H. Holobaca, J. Kyselý, J. Madureira, J. Schwartz, J. J. K. Jaakkola, K. Katsouyanni, M. Hurtado Diaz, M. S. Ragettli, M. Hashizume, M. Pascal, M. de Sousa Zanotti Stagliorio Coélho, N. V. Ortega, N. Ryti, N. Scovronick, P. Michelozzi, P. M. Correa, P. Goodman, P. H. Nascimento Saldiva, R. Abrutzky, S. Osorio, T. N. Dang, V. Colistro, V. Huber, W. Lee, X. Seposo, Y. Honda, Y. L. Guo, M. L. Bell, Y. Guo, Global, regional, and national burden of mortality associated with short-term temperature variability from 2000–19: A three-stage modelling study. *Lancet Planetary Health* **6**, E410–E421 (2022).

53. EPA, Supplementary Material for the Regulatory Impact Analysis for the Supplemental Proposed Rulemaking, "Standards of Performance for New, Reconstructed, and Modified Sources and Emissions Guidelines for Existing Sources: Oil and Natural Gas Sector Climate Review" (2022); `https://t.co/QllVBWDGmL`.

54. K. Rennert, F. Errickson, B. C. Prest, L. Rennels, R. G. Newell, W. Pizer, C. Kingdon, J. Wingenroth, R. Cooke, B. Parthum, D. Smith, K. Cromar, D. Diaz, F. C. Moore, U. K. Müller, R. J. Plevin, A. E. Raftery, H. Ševčíková, H. Sheets, J. H. Stock, T. Tan, M. Watson, T. E. Wong, D. Anthoff, Comprehensive evidence implies a higher social cost of CO_2. *Nature* **610**, 687–692 (2022).

55. N. Vassilieff, N. Rosencher, D. I. Sessler, C. Conseiller, Shivering threshold during spinal anesthesia is reduced in elderly patients. *Anesthesiology* **83**, 1162–1166 (1995).

56. C. H. Saely, K. Geiger, H. Drexel, Brown versus white adipose tissue: A mini-review. *Gerontology* **58**, 15–23 (2011).

57. A. Zanobetti, M. S. O'Neill, C. J. Gronlund, J. D. Schwartz, Susceptibility to mortality in weather extremes: Effect modification by personal and small area characteristics in a multi-city case-only analysis. *Epidemiology* **24**, 809–819 (2013).

58. N. Leigh-Hunt, D. Bagguley, K. Bash, V. Turner, S. Turnbull, N. Valtorta, W. Caan, An overview of systematic reviews on the public health consequences of social isolation and loneliness. *Public Health* **152**, 157–171 (2017).

59. P. Soriano-Hernandez, A. Mejia-Montero, D. van der Horst, Characterisation of energy poverty in Mexico using energy justice and econophysics. *Energy Sustain. Dev.* **71**, 200–211 (2022).

60. B. Falk, R. Dotan, Children's thermoregulation during exercise in the heat—A revisit. *Appl. Physiol. Nutr. Metab.* **33**, 420–427 (2008).

61. Z. Xu, R.A. Etzel, H. Su, C. Huang, Y. Guo, S. Tong, Impact of ambient temperature on children's health: A systematic review. *Environ. Res.* **117**, 120–131 (2012).

62. J. J. Brown, M. Pascual, M. C. Wimberly, L. R. Johnson, C. C. Murdock, Humidity–The overlooked variable in the thermal biology of mosquito-borne disease. *Ecol. Lett.* **26**, 1029–1049 (2023).

63. J. G. Zivin, J. Shrader, Temperature extremes, health, and human capital. *Future of Children* **26**, 31–50 (2016).

64. F. Pavanello, E. de Cian, M. Davide, M. Mistry, T. Cruz, P. Bezerra, D. Jagu, S. Renner, R. Schaeffer, A. F. P. Lucena, Air-conditioning and the adaptation cooling deficit in emerging economies. *Nat. Commun.* **12**, 6460 (2021).

65. T. Kjellström, N. Maître, C. Saget, M. Otto, T. Karimova, *Working on a Warmer Planet: The Impact of Heat Stress on Labour Productivity and Decent Work* (ILO, 2019).

66. H. K. Green, O. Lysaght, D. D. Saulnier, K. Blanchard, A. Humphrey, B. Fakhruddin, V. Murray, Challenges with disaster mortality data and measuring progress towards the implementation of the sendai framework. *Int. J. Disaster Risk Sci.* **10**, 449–461 (2019).

67. International Labour Organization, ILO modelled estimates database (2024); `https://ilostat.ilo.org/data/` [accessed 7 February 2024].

68. OSHA, Heat injury and illness prevention in outdoor and indoor work settings. *Federal Register* (2024).

69. V. Gaigbe-Togbe, L. Bassarsky, D. Gu, T. Spoorenberg, L. Zeifman, World Population Prospects 2022 (Department of Economic and Social Affairs, Population Division, 2022).

70. United Nations Department of Economic and Social Affairs, *World Population Prospects 2022: Summary of Results* (United Nations, 2023).

71. E. M. Fischer, R. Knutti, Robust projections of combined humidity and temperature extremes. *Nat. Clim. Chang.* **3**, 126–130 (2013).

72. J. Yuan, M. L. Stein, R. E. Kopp, The evolving distribution of relative humidity conditional upon daily maximum temperature in a warming climate. *J. Geophys. Res. Atmos.* **125**, e2019JD032100 (2020).

73. J. R. Buzan, K. Oleson, M. Huber, Implementation and comparison of a suite of heat stress metrics within the Community Land Model version 4.5. *Geosci. Model Dev.* **8**, 151–170 (2015).

74. A. Barreca, K. Clay, O. Deschenes, M. Greenstone, J. S. Shapiro, Adapting to climate change: The remarkable decline in the US temperature-mortality relationship over the twentieth century. *J. Political Econ.* **124**, 105–159 (2016).

75. J. Shrader, L. Bakkensen, D. Lemoine, Fatal errors: The mortality value of accurate weather forecasts (IZA Discussion Paper 16253, 2023), p. 1–41; https://papers.ssrn.com/sol3/papers.cfm?abstract_id=4490162.

76. W. Lai, S. Li, Y. Liu, P. J. Barwick, Adaptation mitigates the negative effect of temperature shocks on household consumption. *Nat. Hum. Behav.* **6**, 837–846 (2022).

77. R. Regules García, A. C. Gomez-Ugarte, H. Zoraghein, L. Jiang, Sub-national population projections for Mexico under the Shared Socioeconomic Pathways (SSPs) in the context of climate change. *Popul. Res. Policy Rev.* **43**, 44 (2024).

78. OMB, *Circular A-4* (2023); www.whitehouse.gov/wp-content/uploads/2023/11/CircularA-4.pdf.

79. HM Treasury, *The Green Book: Appraisal and Evaluation in Central Government* (HM Treasury, 2022); www.gov.uk/government/publications/the-green-book-appraisal-and-evaluation in-central-governent (2023).

80 F. C. Lab and C. for International Earth Science Information Network CIESIN Columbia University, High Resolution Settlement Layer (HRSL), 2016, Source imagery for HRSL © 2016 DigitalGlobe 2016.

81. F. E. Harrell, *Regression Modeling Strategies: With Applications to Linear Models, Logistic and Ordinal Regression, and Survival Analysis* (Springer International Publishing, 2015).

82. L. Bergé, Efficient estimation of maximum likelihood models with multiple fixed-effects: The R package FENmlm. CREA Discussion Papers (2018).

83. J. Dong, S. Bronniman, T. Hu, Y. Liu, J. Peng, GSDM-WBT: Global station-based daily maximum wet-bulb temperature data for 1981-2020. *Earth Syst. Sci. Data* **14**, 5651–5664 (2022).

84. K. Sims, A.Reith, E. Bright, J. Kaufman, J. Pyle, J. Epting, J. Gonzales, D. Adams, E. Powell, M. Urban, A. Rose, LandScan Global 2022 (2023); https://landscan.ornl.gov.
